# A Standardized Classification Scheme for Gastroduodenal Disorder Evaluation Using the Gastric Alimetry System: Prospective Cohort Study

**DOI:** 10.1016/j.gastha.2024.09.002

**Published:** 2024-09-07

**Authors:** Chris Varghese, Gabriel Schamberg, Emma Uren, Stefan Calder, Mikaela Law, Daphne Foong, Vincent Ho, Billy Wu, I-Hsuan Huang, Peng Du, Thomas Abell, Charlotte Daker, Christopher N. Andrews, Armen A. Gharibans, Gregory O’Grady

**Affiliations:** 1 Department of Surgery, The University of Auckland, Auckland, New Zealand; 2 Alimetry Ltd, Auckland, New Zealand; 3 School of Medicine, Western Sydney University, Sydney, Australia; 4 Translational Research Center for Gastrointestinal Disorders (TARGID), University of Leuven, Leuven, Belgium; 5 Auckland Bioengineering Institute, The University of Auckland, Auckland, New Zealand; 6 Division of Gastroenterology, University of Louisville, Louisville, Kentucky; 7 Department of Gastroenterology, University of Calgary, Calgary, Canada

**Keywords:** Gastroenterology, Gastroduodenal, Biomarker, Phenotyping, Functional Dyspepsia

## Abstract

**Background and Aims:**

Gastric Alimetry™ (Alimetry, New Zealand) is a new clinical test for gastroduodenal disorders involving simultaneous body surface gastric electrical mapping and validated symptom profiling. Studies have demonstrated a range of distinct pathophysiological profiles, and a classification scheme is now required. We used Gastric Alimetry spectral and symptom profiles to develop a mechanism-based test classification scheme, then assessed correlations with symptom severity, psychometrics, and quality of life.

**Methods:**

We performed a multicenter prospective cohort study of patients meeting the Rome IV criteria for functional dyspepsia and chronic nausea and vomiting syndromes. Patients underwent Gastric Alimetry profiling, and a standardized digital classification framework was devised and applied to separate patients into those with a) abnormal spectral analyses (ie aberrant gastric frequencies, amplitudes, and rhythms); and normal spectral analyses with b) symptoms *correlated* to gastric amplitude (subgroups: sensorimotor, postgastric, and activity-relieved), and c) symptoms *independent* of gastric amplitude (subgroups: continuous, meal-relieved, meal-induced).

**Results:**

Two hundred ten patients were included (80% female, median age 37), of whom 169 met the criteria for chronic nausea and vomiting syndromes and 206 met the criteria for functional dyspepsia (79% meeting both criteria). Overall, 83% were phenotyped using the novel scheme, with 79/210 (37.6%) classified as having a spectral abnormality. Of the remainder, the most common phenotypes were “continuous pattern” (37, 17.6%), “meal-induced pattern” (28, 13.3%), and “sensorimotor pattern” (15, 7.1%). Symptom patterns independent of gastric amplitude were more strongly correlated with depression and anxiety (Patient Health Questionnaire 2: exp(β) 2.38, *P* = .024, State-Trait Anxiety Inventory Short-Form score: exp(β) 1.21, *P* = .021).

**Conclusion:**

A mechanistic classification scheme for assessing gastroduodenal disorders is presented. Classified phenotypes showed independent relationships with symptom severity, quality of life, and psychological measures. The scheme is now being applied clinically and in research studies.

## Introduction

Chronic gastroduodenal symptoms afflict >10% of the global population and impart significant quality of life, economic and health-care burdens.^1^, ^2^, ^3^ Current paradigms rely mainly on symptom-based criteria which group nausea, vomiting, abdominal pain, epigastric burning, early satiation, and excessive fullness into disorders such as chronic nausea and vomiting syndromes (CNVS), functional dyspepsia (FD), and when gastric emptying is delayed, gastroparesis.^4^ However, these classifications overlap,^5^, ^6^, ^7^, ^8^, ^9^ limiting their ability to inform individual patient management. Additional biomarkers and classification schemes are needed to guide differentiated management according to underlying disease mechanisms.

Recently, an approach for advancing biomarker-driven evaluation in these disorders has emerged through Gastric Alimetry™ (Alimetry, New Zealand), which encompasses body surface gastric mapping (BSGM) and simultaneous validated symptom profiling.^5^^,^^10^, ^11^, ^12^ Specific underlying subgroups have recently been revealed through BSGM; for example, nearly a third of patients with CNVS showed spectral abnormalities with aberrant gastric rhythms and low amplitudes identified as a biomarker of gastric neuromuscular dysfunction.^5^ Time-of-test symptom profiles are registered simultaneously through the Gastric Alimetry App, employing standardized pictograms,^13^ revealing additional insights to the relationship between symptoms, a test meal, and gastric electrical responses.^14^^,^^15^ Biomarker-driven phenotyping coupled to simultaneous symptom profiling could therefore offer new modes of disease classification in gastroduodenal disorders, with direct reference to underlying pathophysiology.^16^^,^^17^

Recent clinical studies applying Gastric Alimetry have demonstrated a range of unique test profiles (phenotypes) among patients with gastroduodenal disorders, long-term diabetes, and postoperative gastric dysfunction.^5^^,^^11^^,^^12^^,^^18^, ^19^, ^20^ However, to date there has been no classification scheme to systematically define these phenotypes according to objective criteria. The aim of this study was therefore to present and clinically evaluate a novel standardized and objective approach to phenotyping patients with gastroduodenal disorders, based on Gastric Alimetry spectral and symptom profiling, and supported by recent literature pertaining to recognized underlying disease mechanisms in these populations.^14^ The novel classification scheme was applied in a large cohort of patients with chronic gastroduodenal symptoms, with correlations assessed between classified phenotypes and symptom severity, psychometrics, and quality of life.

## Methods

This was a prospective observational cohort study conducted in Auckland (New Zealand), Calgary (Canada), and Western Sydney (Australia) (Ethical approvals: AH1130, REB19-1925, and H13541). All patients provided informed consent. The study is reported in accordance with the STROBE statement.^21^

### Inclusion Criteria

Patients aged ≥16 years meeting the clinical criteria for CNVS, FD, or both per the Rome IV criteria were eligible for inclusion.^4^ Patients were recruited by gastroenterologists, and all were clinically evaluated to rule out alternative causes for symptoms. Exclusion criteria were metabolic, neurogenic, or endocrine disorders known to cause gastric dysmotility other than diabetes (eg, scleroderma, multiple sclerosis, and hyperthyroidism), an active gastrointestinal infection (including Helicobacter pylori), previous gastric or esophageal surgery, history of gastrointestinal malignancy, or current pregnancy. Gastric emptying status was not a study exclusion criterion. Patients with cyclical vomiting syndrome or cannabinoid hyperemesis were excluded. Specific exclusion criteria related to Gastric Alimetry were active abdominal wounds or abrasions, fragile skin, and allergies to adhesives. Previous investigations have identified overlapping pathological changes in gastroparesis and CNVS, such that they may lie on the same disease spectrum^22^, ^23^, ^24^, ^25^, ^26^; we have therefore included patients that meet the Rome IV criteria with or without gastroparesis in line with previous publications.^5^^,^^27^ Gastric emptying testing results were unavailable in this cohort.

### Study Protocol

**Figure 1:**
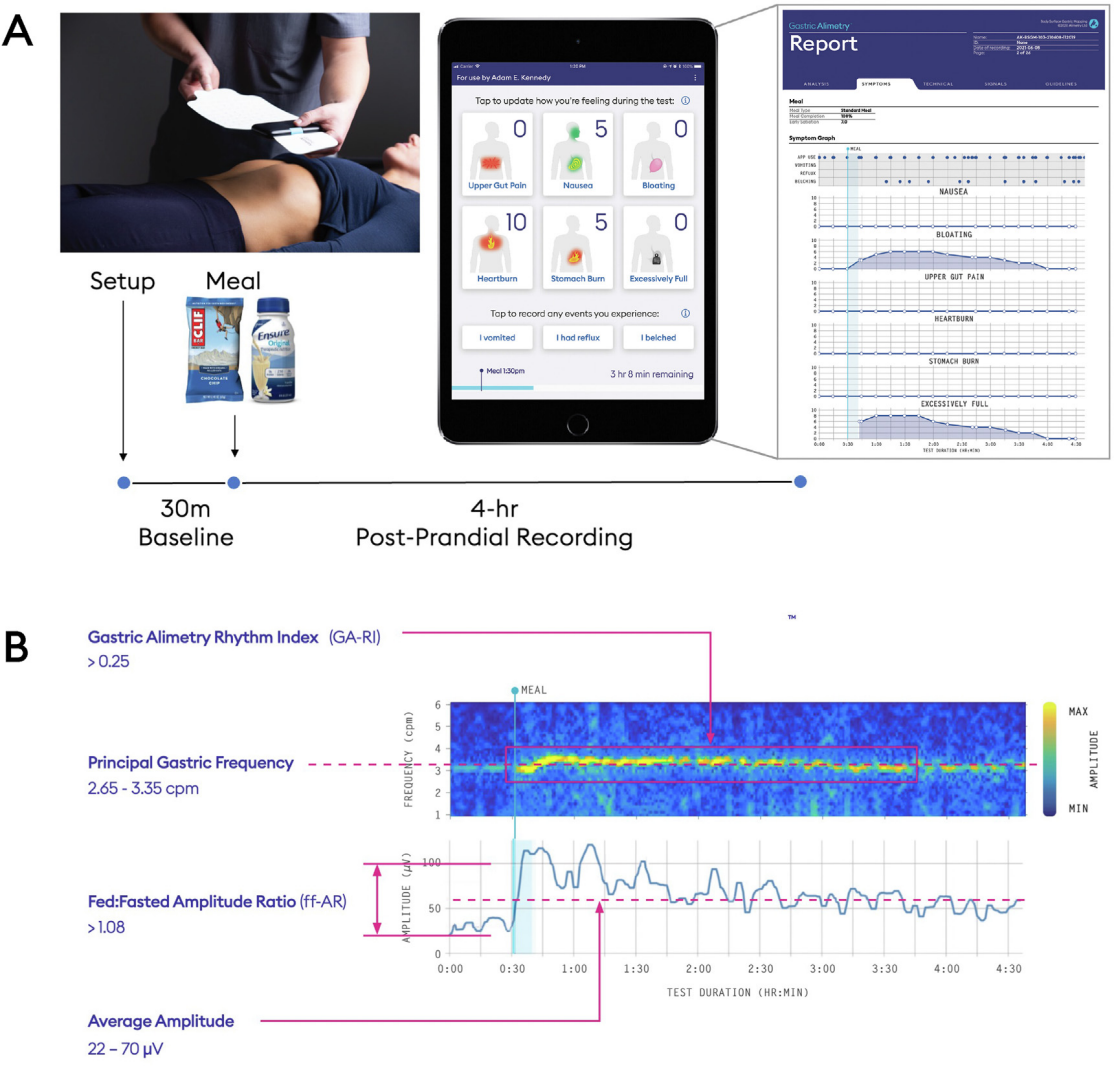
Summary of Gastric Alimetry™ test. A: Gastric Alimetry test protocol encompassing 30-minute fasting, 10-minute meal stimulus and 4-hour postprandial recording with concurrent symptom logging at 15-minute intervals. B: Validated reference intervals for spectral metrics from body surface gastric mapping.^29^

The Gastric Alimetry System encompasses a high-resolution stretchable electrode array (8 x 8 electrodes; 20-mm interelectrode spacing; 196 cm^2^), a wearable Reader, an iPadOS App for set-up, anthropometric measurement–based array placement, concurrent validated symptom profiling during the test, and a cloud-based analytics and reporting platform (Figure 1).^5^^,^^13^^,^^28^ The standard Gastric Alimetry test protocol was followed.^28^ Participants fasted for >6 hours and avoided caffeine and nicotine before testing. Array placement was preceded by shaving if necessary, and skin preparation (NuPrep; Weaver & Co, CO, USA). Recordings were performed for a fasting period of 30 minutes, followed by a 482-kCal meal consumed over 10 minutes and a 4-hour postprandial recording. The meal consisted of an oatmeal energy bar (250 kcal, 5 g fat, 45 g carbohydrate, 10 g protein, 7 g fiber; Clif Bar & Company, CA, USA) and Ensure (232 kcal, 250 mL; Abbott Nutrition, IL, USA). Diabetic, gluten-free, and vegan options of similar caloric load were also available. Participants sat reclined in a chair and were asked to limit movement, talking, and sleeping, but were able to read, watch media, work on a mobile device, and mobilize for comfort breaks.

Patient-reported symptoms were captured using Gastric Alimetry’s validated symptom-logging app.^13^^,^^28^ Patients logged symptoms including upper gut pain, nausea, bloating, heartburn, stomach burn, and excessive fullness with the aid of pictograms at 15-minute intervals and could indicate discrete events including vomiting, reflux, and belching when they occurred. Symptom severity was assessed via an 11-point likert scale (0 indicating ‘no symptom;’ 10 indicating the ‘worst symptom imaginable’).

### BSGM Methodology

Gastric Alimetry’s BSGM system is a form of high-resolution electrogastrography which registers gastric bioelectrical slow-wave activity as well as gastric contractile activity through an increase in signal power.^28^^,^^30^ Spectral analysis (Figure 1B) of BSGM data represents the frequency, rhythm, and amplitude characteristics of the underlying slow waves that coordinate gastric motility. Normative reference intervals have been validated for the body mass index (BMI)-adjusted amplitude, a measure of active electrophysiological activity within the gastric frequency range; principal gastric frequency, the frequency in cycles per minute, of gastric slow waves as registered on the body surface; Gastric Alimetry Rhythm Index (GA-RI), a measure of the stability and consistency of gastric slow waves; and the fed-fasted amplitude ratio, a measure of the postprandial increase in gastric amplitude.^29^

### Classification Scheme

**Figure 2:**
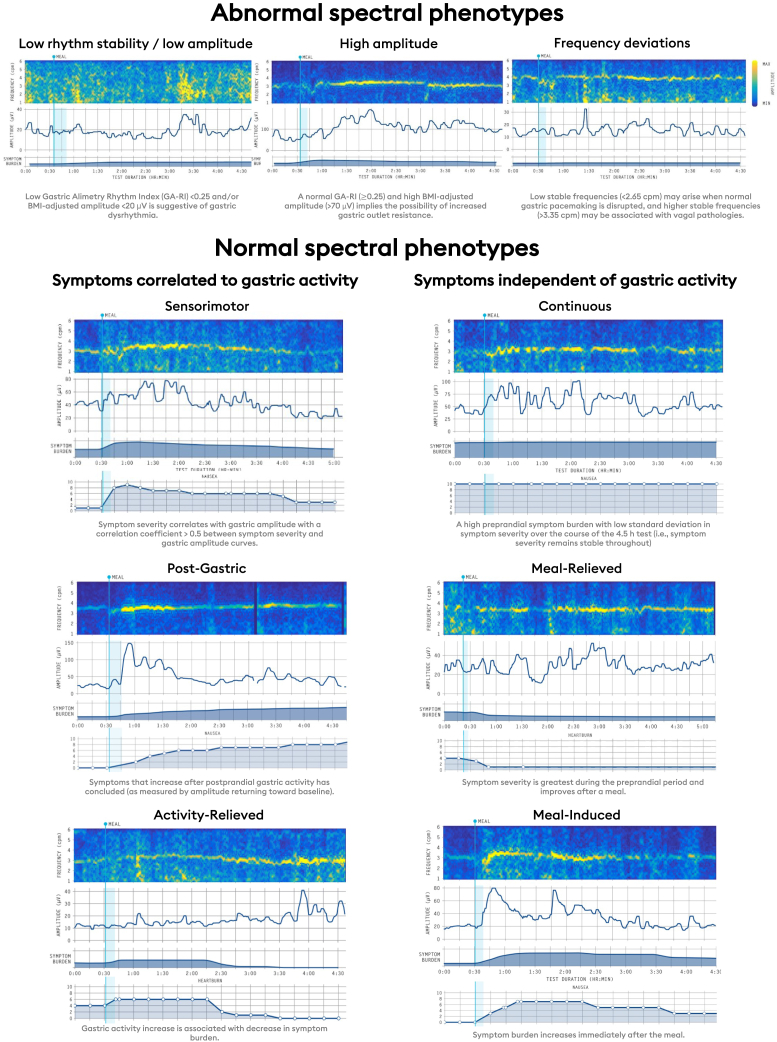
Data arising from the Gastric Alimetry™ test are then synthesized into a novel classification scheme including abnormal spectral analysis, symptom profiles aligned to the gastric amplitude curve,^14^ and symptom profiles independent of the gastric amplitude curve. Representative examples of each phenotype are presented.

A classification scheme was derived on the basis of a comprehensive review and expert consensus.^14^^,^^31^ Three groups were defined; those with spectral abnormalities, symptom profiles *correlated* with the gastric amplitude curve, and symptom profiles *independent* of the gastric amplitude curve. An outline of the classification scheme with representative cases is shown in Figure 2, and is detailed below together with physiological and mechanistic considerations (see further details in Supplementary Methods).^14^^,^^31^

#### Spectral phenotypes

Three key categories of spectral phenotypes were defined on the basis of a comprehensive technical review of the electrophysiology literature and are represented with validated reference intervals:^14^^,^^29^

• **Low rhythm stability/low amplitude**: A low GA-RI <0.25 and/or BMI-adjusted amplitude <20 μV is suggestive of sustained gastric dysrhythmia and/or neuromuscular dysfunction.^5^
• **High stable amplitude**: A normal GA-RI (≥0.25) and high BMI-adjusted amplitude (>70 μV), which is hypothesized to indicate the possibility of increased gastric outlet resistance.^32^
• **Isolated frequency deviations**: Low stable frequencies (<2.65 cpm), most commonly arising when normal gastric pacemaking is disrupted (eg after gastric surgery and gastroparesis)^33^ and higher stable frequencies (>3.35 cpm), most commonly arising in association with diabetic neuropathy and vagal injury.^12^^,^^18^

#### Symptom phenotypes, *correlated* with gastric activity

When the spectrogram was normal, 3 patterns of symptoms were described based on their relation to the gastric amplitude curve (sensorimotor, postgastric, and activity-relieved (long-lag)):^34^

• **Sensorimotor**: Symptom severity correlates with gastric amplitude (defined as correlation coefficient > 0.5 between individual symptom severity and gastric amplitude curves). This correlation between gastric activity and individual symptoms are hypothesized to primarily reflect visceral hypersensitivity and/or disordered accommodation.^35^
• **Postgastric**: Symptoms that increase after postprandial gastric activity has concluded (as measured by amplitude returning toward baseline or a downslope as symptoms upslope). This was defined on the basis of average differences between cumulative sums of normalized symptom and amplitude curves ie, −1 indicates all symptoms occurring before all gastric activity and +1 all symptoms occurring after gastric activity (see Schamberg et al., for further details^34^). This category is hypothesized to primarily reflect symptoms arising from the small bowel ie, that arise after gastric emptying has progressed.^36^
• **Activity-relieved (long-lag)**: Gastric activity increase is associated with decrease in symptom burden (ie, an upslope in gastric activity with a downslope in symptoms). This reflects the opposite direction of effect when comparing the cumulative distribution functions of symptom and amplitude curves to that of the postgastric phenotype. The spectrogram in this phenotype typically demonstrates a long lag between the meal and the onset of a gastric meal response, which is hypothesized to relate to postprandial gastric dysfunction plausibly in association with maldistribution of intragastric contents.^37^

#### Symptom phenotypes, *independent* of gastric activity

Three patterns of symptoms were described based on an increase, decrease, or constant severity of symptoms in relation to a meal stimulus and independent of the gastric amplitude curve. These phenotypes, having normal spectrograms and being independent of gastric amplitude, were hypothesized to relate to disordered gut–brain interactions, systemic inflammation,^38^^,^^39^ or as yet unknown factors.^4^

• **Continuous**: A high preprandial symptom burden with low standard deviation in symptom severity over the course of the 4.5-hour test (ie, symptom severity remains stable throughout).^5^^,^^14^ This is supported by the presence of symptoms in the preprandial period with only modest exacerbation with a meal, and which do not resolve when gastric amplitude returns toward baseline, suggesting a symptom origin not related to gastric motility (technical details on the objective quantification of this pattern is defined in Schamberg et al^34^).
• **Meal-relieved**: Symptom severity is greatest during the preprandial period and improves after a meal. It is defined as the negative change in average symptom severity between the preprandial period and first postprandial hour.
• **Meal-induced**: Symptom burden increases immediately after the meal. It is defined as the positive change in average symptom severity between the preprandial period and first postprandial hour, but without meeting the criteria for the sensorimotor phenotype above.

Objective identification of these symptom profiles have recently been demonstrated in a large database using an automated algorithm.^34^ In practice, a patient may meet the criteria for more than 1 pattern (eg a spectral abnormality and a symptom phenotype), and/or patients may have different patterns for different symptoms. A hierarchy was therefore instituted such that if any test met 1 of the above criteria, they were phenotyped per a prioritization as follows: 1) spectral abnormality, 2) symptom profiles correlated to gastric activity (a. sensorimotor, b. postgastric, c. activity-relieved), and 3) symptoms profiles independent of gastric activity (a. continuous, b. meal-relieved, c. meal-induced). This prioritization was chosen based on preferring the most objective pathophysiological justification for phenotyping in any given case. Symptom profiles were also applied to ‘overall’ curves, defined as the average of the curves associated with each symptom.

### Patient-Reported Outcomes

Patients were classified based on the self-reported responses to Rome IV criteria for FD and CNVS.^4^ Psychological factors included depression as measured by the Patient Health Questionnaire 2,^40^ and state anxiety as measured by the state subscale of the State-Trait Anxiety Inventory Short-Form score.^41^ Long term symptoms were measured by the Patient Assessment of Upper Gastrointestinal (PAGI) Symptom Severity Index (PAGI-SYM)^42^ and Gastroparesis Cardinal Symptom Index.^43^ Quality of life was measured by the PAGI Disorders-Quality of Life (PAGI-QOL)^44^ and the EuroQoL-5D-5L (EQ-5D).^45^ Due to a change in psychometrics used in our prospective database, some missing data exist which were addressed with pairwise elimination as the random nature of missingness could not be confirmed to meet the assumptions of imputation.

### Analysis

Artifacts were automatically detected and rejected where possible using the validated Gastric Alimetry algorithm (v 2.4.1).^46^ Tests with >1 hour of missing signal data were excluded. All analyses were performed in Python v3.9.7 and R v.4.0.3 (R Foundation for Statistical Computing, Vienna, Austria). Rates are reported as n (%). Descriptive statistics were performed using one-way analysis of variance, independent samples t test, Mann-Whitney U, Kruskal–Wallis test, for continuous variables and chi-square tests or Fisher’s exact test as appropriate. Multivariable linear regressions were performed to assess associations between symptom profiles (described above) and psychometrics (Patient Health Questionnaire 2, State-Trait Anxiety Inventory Short-Form), gastroduodenal symptom severity (PAGI-SYM), and quality of life (PAGI-QOL and EQ-5D) with reference to uncategorized (‘Other’). These associations were adjusted for age, sex, and BMI, and outputs were reported as exponentiated beta coefficient and 95% confidence intervals. Significantly skewed outcome variables were log transformed, and normality assessments were made through inspection of Q-Q plots.

## Results

### Demographics of Cohort

A cohort of 210 participants meeting the Rome IV criteria for a chronic gastroduodenal disorder was recruited. Of this cohort, 169 (80%) were female, median age was 37 (interquartile range [IQR] 25–54), the median BMI was 24.1 kg/m^2^ (IQR 14.8–29.8), and 31 (14.8%) had diabetes mellitus. Median meal completion during Gastric Alimetry™ testing was 100% (IQR 70–100).

### ROME IV Criteria

Overall, 169 (80%) met the Rome IV criteria for CNVS and 206 (98%) for FD. Of those who met the criteria for FD, 171 (81%) had the postprandial distress (PDS) subtype, while 175 (83%) had the epigastric pain syndrome (EPS) subtype. Notably, as seen in Figure A1 and A2, disorders defined by Rome IV in the cohort were substantially overlapping, with 79% meeting both the CNVS and FD criteria. Overall, 59% had overlap of CNVS and both PDS and EPS subtypes of FD. Similarly, among those with FD alone, 68.0% met the criteria for both the PDS and EPS subtypes. Only 4 of 169 (2.4%) of those that met the criteria for CNVS did not have overlapping FD. Generally, greater overlap of Rome IV disorders meant greater symptom burden (Figure S1 and Table A1). Average burden of each continuous symptom (upper gut pain, nausea, bloating, heartburn, stomach burn, and excessive fullness) are compared in Figure A3A-F for the Rome IV diagnostic categories and phenotypic categories of the novel classification scheme.

### Spectral Phenotypes

**Figure 3:**
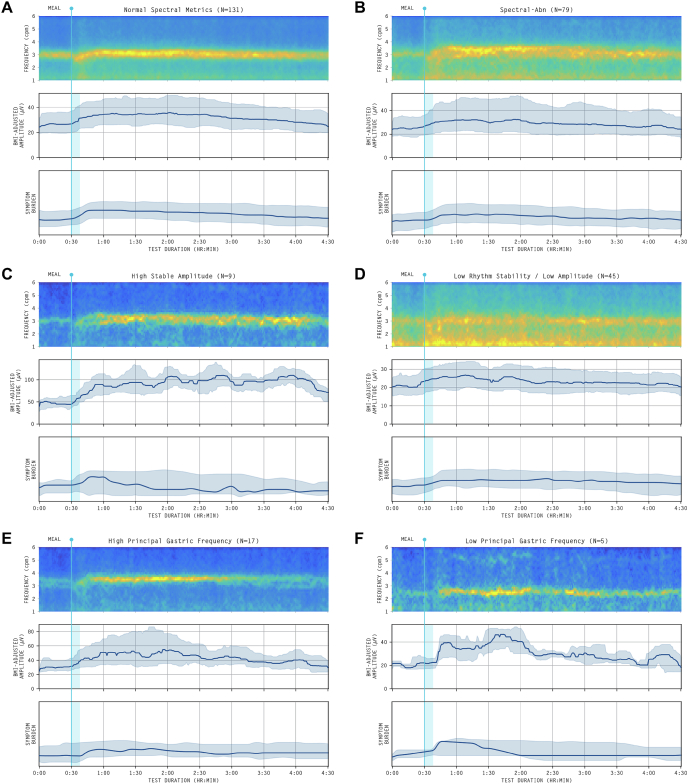
Average spectrograms (frequency-amplitude graphs) showing frequencies on a scale from low power (dark blue) to high power (bright yellow), indicating gastric meal responses and rhythm, and median (IQR shaded) BMI-Adjusted Amplitude and symptom burden. The meal time and duration are indicated by a vertical blue bar at 30 min. A) normal spectral data, B) any abnormal spectral metrics, C) high stable amplitude, D) low rhythm stability/low amplitude, E) high principal gastric frequency, F) low principal gastric frequency.

Overall, 79 (37.6%) patients had an abnormal spectrogram, defined per the normative reference intervals in Figure 1B, which were distributed in the following groups (average spectrograms for each group are provided in Figure 3, with further data provided in Table A2): *Low rhythm stability/low amplitude*: 48 (22.9%) met the criteria for low rhythm stability and/or low amplitude. Of these, 36 had a low rhythm stability only, 4 had both a low rhythm stability and low amplitude, and 8 had a low amplitude only. *High stable amplitude*: 12 (5.7%) had normal GA-RI and high BMI-adjusted amplitude. *Isolated frequency deviations*: 7 (3.3%) had a low frequency and 21 (10%) had a high frequency. There was a substantially higher frequency of diabetics among patients with high stable frequency spectrograms than without (9 of 21, 42.9% vs 22 of 189, 11.6%; *P* < .001). After applying our hierarchal classification scheme, 48 (22.9%) had low rhythm stability/low amplitude, 9 (4.3%) had high stable amplitude, 5 (2.4%) had a low stable frequency, and 17 (8.1%) had a high stable frequency.

**Figure 4:**
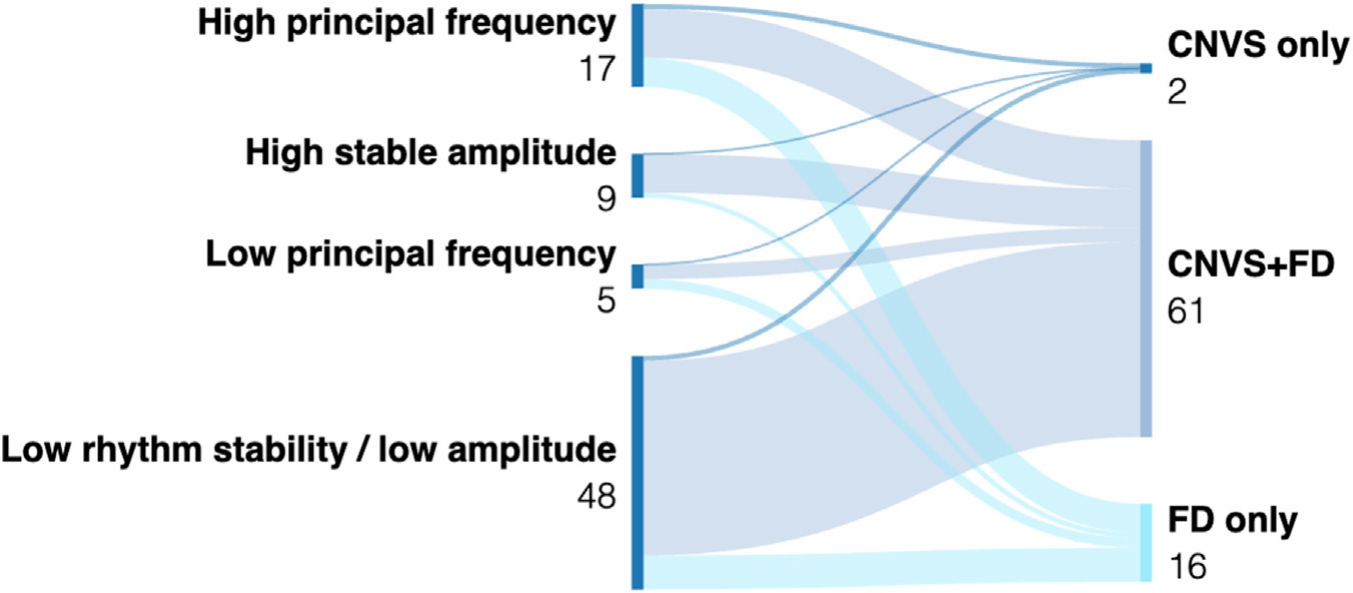
Sankey plot of spectral phenotypes in relation to the Rome IV criteria.

There was no correlation between patients having spectral abnormalities and Rome IV diagnostic categories, as shown in the Sankey plot in Figure 4. Of the 41 with FD only, 16 (39%) had spectral abnormalities, 2 (4.9%) had amplitude-related symptoms, and 12 (29.3%) had amplitude-independent symptoms. Of the 165 with CNVS and FD overlap; 61 (37%) had spectral abnormalities, 25 (15.2%) had amplitude-related symptoms, and 56 (33.9%) had amplitude-independent symptoms. Two of 4 (50%) of those with CNVS only had spectral abnormalities.

### Symptom Phenotypes, *Correlated* with Gastric Activity

#### Sensorimoto

**Figure 5:**
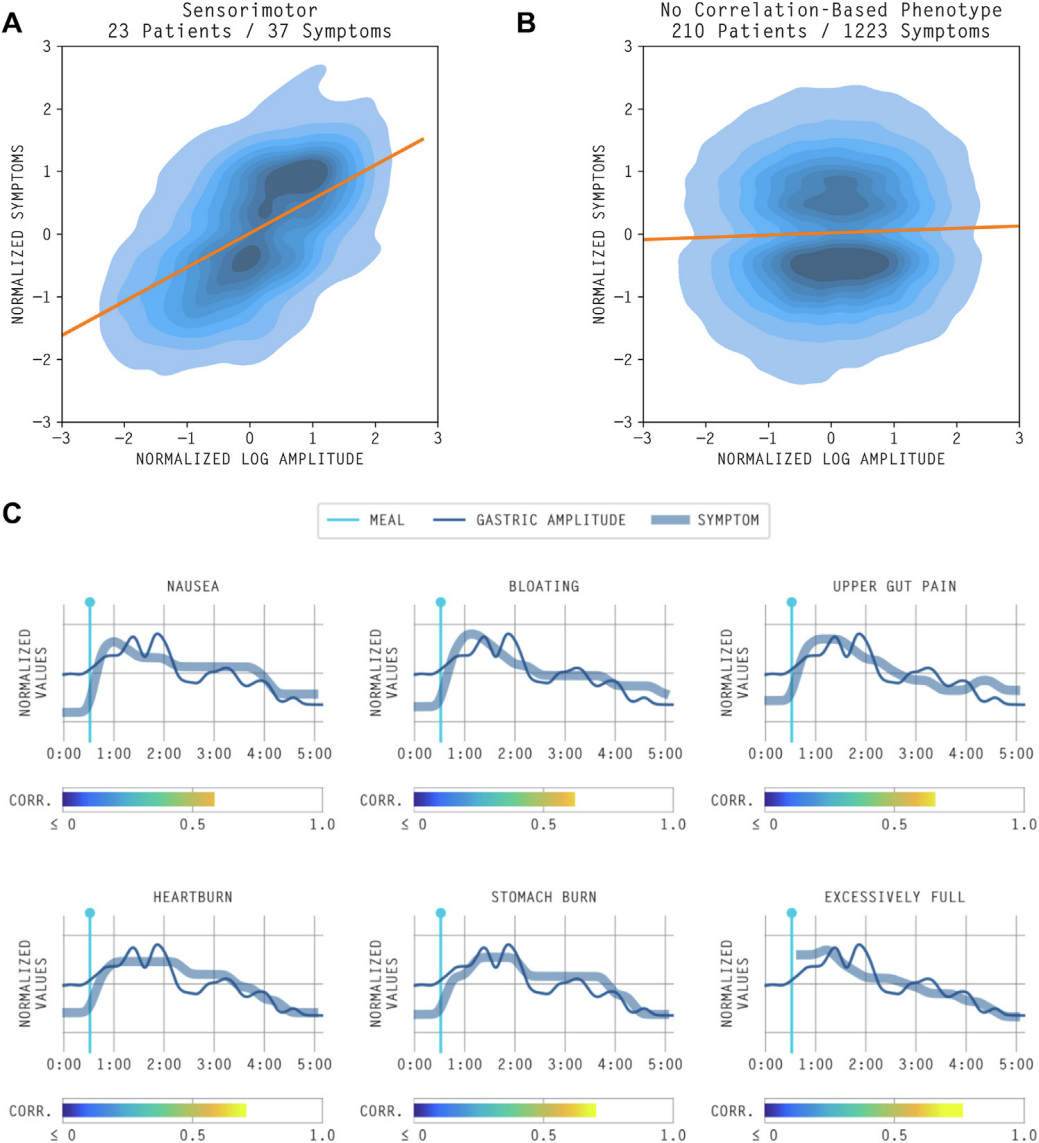
Density plot of normalized amplitude and symptom burden visualizing A) sensorimotor (r = 0.54, *P* < .001) vs B) nonsensorimotor (r = 0.036, *P* < .001) pattern profiles. C) Normalized average symptom burden and gastric amplitude curves for an example of sensorimotor symptom profiles.

13 (6.2%) total average symptom burden curves followed a sensorimotor pattern. Individually, 15 (7.1%) bloating curves followed a sensorimotor pattern, 8 (3.8%) nausea curves, 6 (2.9%) stomach burn curves, and 5 (2.4%) upper gut pain curves. The average symptom-amplitude correlation was r = 0.62 (standard deviation [SD] 0.016) for all sensorimotor curves. Only 3 heartburn curves followed a sensorimotor pattern. The normalized average symptom profile as it relates to the normalized gastric amplitude for an example of the sensorimotor pattern is depicted in Figure 5.

#### Postgastric

The postgastric pattern was relatively uncommon, occurring in 9 symptom curves across 9 patients. An example of the postgastric pattern is seen in Figure A4. This pattern was seen predominantly in curves for upper gut pain (n = 3 of 9; 33%) and bloating (n = 2 of 9; 22%) symptoms. The mean symptom/amplitude time lag was 0.38 (SD 0.12; ie, most symptoms occur after gastric activity; Figure A4).

#### Activity-relieved

The activity-relieved pattern was seen in 15 symptom curves across 11 patients (Figure A4B). The mean symptom/amplitude time lag was −0.30 (SD 0.10). A visual depiction of the difference between cumulative distribution functions of the lag-based and nonlag phenotypes is seen in Figure A4C-E.

### Symptom Phenotypes, *Independent* of Gastric Activity

#### Continuous

This was the most common single phenotype observed after spectral abnormalities. Forty-eight (21.4%) total average symptom curves followed a continuous pattern. Individually, 27 (12.9%) nausea curves, 37 (17.6%) bloating curves, 9 (4.3%) heartburn curves, 16 (7.6%) stomach burn curves, 30 (14.3%) upper gut pain curves, and 40 (19.0%) excessive fullness curves followed a continuous pattern. Among all curves following a continuous pattern, the average symptom burden was 4.3 of 10 (SD 0.77; Figure A5A) and the average range of symptoms was 1.77 (SD 0.57; Figure A5A).

#### Meal-relieved

The meal-relieved pattern was seen in 8 symptom curves across 8 patients (Figure A5B). Among all curves following a meal-relieved pattern, the average meal-induced symptom change was −3.65 (SD 1.54).

#### Meal-induced

Twenty-four (11.4%) total average symptom curves followed a meal-induced pattern. Individually, 45 (21.4%) nausea curves, 48 (22.9%) bloating curves, 13 (6.2%) heartburn curves, 20 (9.5%) stomach burn curves, and 32 (15.2%) upper gut pain curves followed a meal-induced pattern. Among all curves following a meal-induced pattern, the average meal-induced symptom change was 2.83 (SD 0.65; Figure A5C).

#### Profile prevalence

Allowing for multiple patterns to be attributed to a symptom curve, the prevalence of patterns by symptom is summarized in Table A3. The prevalence of each symptom profile across different Rome IV disorders is shown in Table A4. The most common symptoms to follow a sensorimotor pattern were bloating (30%) and stomach burn (16%); meal-induced symptoms: bloating (26.4%) and stomach burn (24.7%); postgastric symptoms: upper gut pain (27.3%) and bloating (18.2%); and activity-relieved symptoms: nausea (25%), stomach burn (25%), and upper gut pain (12.5%).

### Clinical Interpretation Framework

**Figure 6:**
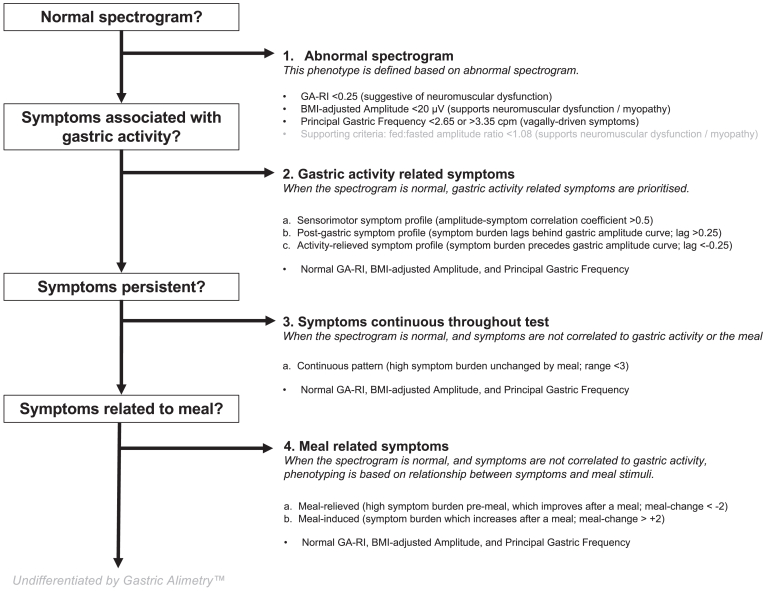
Gastric Alimetry™ flow diagram for incorporation of symptom profiles in a hierarchical classification schema.

The proposed hierarchical classification schema outlined in Figure 6 was next applied to this cohort. As per the first level of the phenotyping hierarchy, 79 (37.6%) of the cohort were classified as having a spectral abnormality.

Of the remaining 131 with a normal spectrogram; 37 (17.6%) had a continuous phenotype, 28 (13.3%) had a meal-induced phenotype, and 15 (7.1%) had a sensorimotor phenotype. Of the other 51 subjects, 8 had an activity-relieved phenotype, 3 had a meal-relieved phenotype, and 4 had a postgastric phenotype. Thereafter, 36 (17.1%) remained undifferentiated such that with a normal spectrogram, their symptom curves did not meet the criteria for a predefined pattern. Therefore, 82.9% of patients were successfully phenotyped in total.

### Clinical Validity with Comparisons to Symptoms, Depression, Anxiety, and Quality of Life

Having a spectral abnormality was strongly associated with daily symptom severity and poorer quality of life (*P* < .05; Table), but not depression or state anxiety (*P* > .05; Table). The symptom profile classifications (per Figures 2 and 6) demonstrated a number of distinct and strong associations with chronic symptom burden, state anxiety, depression, and quality of life (see Table). Having a spectral abnormality or symptoms correlated to the gastric amplitude (eg, sensorimotor, postgastric, or activity-relieved phenotypes) were associated with symptom severity on the validated survey instruments (exp(β) >1.5, *P* < .05; Table and A5), but not anxiety or depression (*P* > .05). Those with symptoms that were independent of the gastric amplitude (eg, continuous, meal-relieved, and meal-induced phenotypes), had the highest symptom severities (exp(β) 1.8, 95% confidence intervals (CI) 1.26–2.59, *P* = .002 for Gastroparesis Cardinal Symptom Index and exp(β) 1.68, 95% CI 1.23–2.31, *P* < .001 for PAGI-SYM), poorest quality of life (exp(β) 0.82, 95% CI 0.74–0.90, *P* = .001 for EQ-5D, and exp(β) 0.52, 95% CI 0.36–0.76, *P* < .001 for PAGI-QOL), with higher depression (exp(β) 2.38, 95% 1.13–5.03, *P* = .024), and state anxiety (exp(β) 1.21, 95% CI 1.03–1.42, *P* = .021) scores. Correlational analyses of individual phenotypes are summarized in Table A5.

**Table:** Association Between Gastric Alimetry™ Classification Categories and Symptoms (GCSI, PAGI-SYM), Depression (PHQ-2), Anxiety (State STAI), and Quality of Life (EQ-5D, PAGI-QOL)

Characteristic	GCSI (n = 209)			PHQ-2 (n = 202)			State STAI (n = 164)			PAGI-QOL (n = 209)			PAGI-SYM (n = 209)			EQ-5D (n = 147)		
	exp(β)	95% CI	p	exp(β)	95% CI	p	exp(β)	95% CI	p	exp(β)	95% CI	p	exp(β)	95% CI	p	exp(β)	95% CI	p
Age	0.99	0.98–0.99	<.001	0.98	0.97–1.00	.031	1	0.99–1.00	.2	1.01	1.00–1.02	.063	0.98	0.98–0.99	<.001	1	1.00–1.00	.5
Sex																		
Female	—	—		—	—		—	—		—	—		—	—		—	—	
Male	0.59	0.44–0.81	.001	0.92	0.49–1.74	.8	1.09	0.95–1.25	.2	1.28	0.93–1.76	.14	0.65	0.50–0.85	.002	1.04	0.96–1.12	.4
BMI	1.02	1.00–1.04	.13	1.03	0.98–1.07	.2	1.01	1.00–1.01	.2	0.99	0.97–1.01	.2	1.02	1.01–1.04	.01	1	0.99–1.00	.4
Gastric Alimetry™ phenotype																		
Reference	—	—		—	—		—	—		—	—		—	—		—	—	
Amplitude-independent	1.8	1.26–2.59	.002	2.38	1.13–5.03	.024	1.21	1.03–1.42	.021	0.52	0.36–0.76	<.001	1.68	1.23–2.31	.001	0.82	0.74–0.90	<.001
Amplitude-related	1.67	1.07–2.62	.026	1.68	0.66–4.25	.3	1.15	0.95–1.40	.2	0.53	0.33–0.85	.009	1.55	1.05–2.29	.03	0.89	0.79–1.00	.061
Spectral-Abn	1.62	1.14–2.30	.008	1.34	0.65–2.76	.4	1	0.86–1.16	>.9	0.68	0.47–0.99	.044	1.54	1.13–2.09	.006	0.92	0.84–1.01	.077

EQ-5D, EuroQoL-5D-5L; GCSI, Gastroparesis Cardinal Symptom Index; PHQ 2, Patient Health Questionnaire 2; State STAI, State-Trait Anxiety Inventory Short-Form.

These findings were independently consistent when evaluating profile patterns for each individual symptom (Table A6). Of note, a continuous pattern of nausea was most strongly associated with depression scores (exp(β) 3.26, *P* = .018). Furthermore, a spectral abnormality was strongly associated with nausea severity (exp(β) 1.43, *P* = .012), but not significantly correlated with depression or anxiety (*P* > .05; Table A6).

## Discussion

The aim of this study was to present and clinically evaluate a novel standardized approach to classifying gastroduodenal disorders through a mechanism-based scheme enabled by the Gastric Alimetry System. We divided patients into 3 groups, comprising those with spectral abnormalities (indicating an objective motility abnormality), those with symptoms correlated with gastric activity, and those with symptoms independent of gastric activity. This new scheme provided a specific and physiologically motivated set of patient classifications,^4^ with the distinct profiles yielding differentiated correlations to chronic symptom severities, psychometrics, and quality of life in a large cohort analysis.

This study advances a provisional phenotyping classification proposed by the BSGM Consortium to a first clinical scheme,^14^ supporting the value of mechanistic symptom profiling when spectral BSGM data are within established normative reference intervals.^29^ A third of patients with CNVS have previously been found to have abnormalities consistent with gastric neuromuscular dysfunction (abnormal rhythm stability and low amplitudes),^5^ which may be associated with pathological injury in interstitial cells of Cajal networks.^24^^,^^25^ Given the substantial overlap between CNVS and FD, as seen in this cohort (79%), we also anticipated neuromuscular pathologies to be a relevant mechanism in a subset of patients with FD symptoms, as recently demonstrated by the NIH Gastroparesis Clinical Research Consortium.^23^ This was demonstrated in our results, which showed similar proportions of spectral abnormalities between Rome IV defined groupings. Spectral abnormalities were present in 38% of our cohort, while a further 45% with normal spectral recordings were then able to be phenotyped into a dominant category based on their symptom profiles, offering a putative mechanism for symptoms in a total of 83% of patients in the cohort.

The novel symptom profile set applied in this work links to several specific mechanisms already known to underlie chronic gastroduodenal symptoms. Firstly, the work further defines the subset of patients where the gut–brain axis dysregulation may be a key driver of gastroduodenal symptomatology, possibly intermediated through systemic inflammation (although it is acknowledged that unknown alternative factors not assessed by this technology may also contribute).^38^^,^^47^^,^^48^ While current paradigms define all Rome IV disorders under the umbrella of ‘disorders of gut–brain interaction’ or DGBIs,^6^^,^^49^^,^^50^ these patients are also known to have heterogeneous underlying pathophysiologies including neuromuscular pathologies, visceral hypersensitivity, accommodation disorders, dysbiosis and immune activation.^22^, ^23^, ^24^, ^25^, ^26^ In this large cohort study, we find that patients with a normal spectrogram, considered to indicate an intact gastric neuromuscular apparatus, and a symptom profile independent of gastric activity (ie, continuous, meal-induced, and meal-relieved) clearly had the strongest correlations with depression and state anxiety scores. Conversely, those with abnormal spectrograms had relatively low depression scores. This finding is emerging as a robust and consistent association, now having been replicated in multiple cohorts of increasing size.^5^^,^^11^^.^^12^ The important clinical implication is that it appears plausible to define significant subsets of patients into primarily centrally mediated vs gastric pathophysiology subgroups, which may allow improved patient selection for principally psychological or central nervous system-targeted therapies vs gastric-targeted therapies such as prokinetics or endoscopic interventions. However, it is also acknowledged that alternative mechanisms may also be contributory, including in the meal-induced symptoms group who show weak correlations with gastric activity. It has been proposed in one study that such symptoms may relate to both gastric and small bowel origins.^7^ Additionally, the relationship between symptoms and psychological factors is understood to be bidirectional, and further work is ongoing to differentiate causal chains of symptom genesis.^51^

In addition, this study introduces a novel approach to localizing the source of symptom genesis through the correlations between specific symptom and gastric amplitude profiles when recorded simultaneously using the Gastric Alimetry System. Symptoms such as pain and burning can be difficult for patients to localize and define, and therefore difficult to assess in a standardized manner, particularly given the complex process of visceral perception.^52^ The temporal data offered by time-of-test symptom profiling can therefore offer more objective data on anatomico-pathological correlation, similar to the widely accepted paradigm of symptom correlations in esophageal pH testing.^53^ We agree with the hypothesis that a meal-induced symptom curve that decays over the 4-hour postprandial period is more likely indicative of a gastric source for symptoms, given symptom burden increases when food contents arrive at the stomach and reduces when food contents transit to the small intestine^14^^,^^31^^,^^36^; whereas a postgastric profile is more suggestive of a small intestinal pathology as symptoms arise after the gastric activity cycle has completed as also reported by Vanheel and colleagues.^14^^,^^36^ The finding that the postgastric pattern was most often associated with bloating and postprandial pain symptoms may also fit with a prominent hypotheses relating to the role of the duodenum in triggering FD symptoms.^54^, ^55^, ^56^

Furthermore, we identify another subgroup in which sensorimotor features may be the dominant disease mechanism, defined on the basis of strong objective correlation between symptoms and gastric amplitude, offering additional potential actionable biomarkers. Sensorimotor symptoms have been described in association with both visceral hypersensitivity or disorders of gastric accommodation.^57^ Part of this subgroup may therefore reflect a cohort of patients with gastric hypersensitivity^58^; which could be explained by a hyper-responsive enteric nervous system to non-noxious stimuli or an exaggeration of an inherent volumetric mechanoreception capacity of the stomach, which may include postinfective disorders and/or accompanying immune activation or neuromuscular injury.^59^, ^60^, ^61^ Gastric sensorimotor dysfunction is a common hypothesis in FD, gastroparesis, CNVS, and dumping syndrome,^35^ and the presented amplitude-coupled symptom burden profile may select for patients where this mechanism is relevant.^17^^,^^62^ Further work is now required to characterize signatures of gastric dysaccommodation in BSGM studies, so that a more specific understanding of this subgroup can be achieved.

Several limitations are noted regarding this study. Some subgroups introduced in this study were relatively uncommon, specifically the meal-relieved, activity-relieved, and postgastric phenotypes, and hence further work is required to validate and assess their significance. For example, the ‘long lag’ observation in the spectrograms of patients with the activity-relieved phenotype could be objectively interrogated against a meal response reference range, to determine if this is a distinct disease biomarker. Furthermore, a minority of patients remained uncharacterized after spectral analyses and symptom profiling was applied; however, additional analytical approaches targeting the spatial propagation of gastric slow waves and the temporal dynamics of the gastric amplitude curve, as it relates to gastric motility, are expected to elucidate further phenotypes in future. Additionally, it is known that disordered fundic accommodation occurs in a fifth of patients with chronic gastroduodenal symptoms possibly comprising another mechanistic phenotype not measured here.^63^ Much like other disease classification systems,^4^^,^^64^ the proposed BSGM classification scheme will be iterated upon to improve patient classification. Additionally, gastric emptying status was not systematically assessed as part of this large cohort of patients from the community. Gastric emptying and BSGM measure different aspects of gastric function (with gastric emptying being insensitive to neuromuscular pathologies), and because gastric emptying is labile over time, we consider it preferable to perform both studies simultaneously in dedicated studies for valid comparisons of the 2 tests.^23^^,^^65^

In conclusion, this study presents and evaluates a novel classification scheme for gastroduodenal disorders, based on Gastric Alimetry testing and proposed underlying disease mechanisms. Given the novel phenotypes demonstrate strong and distinctive correlations with chronic symptom severity, psychological factors, and quality of life, they offer a useful adjunct in the diagnostic pathways of patients with chronic gastroduodenal symptoms. The scheme is now being applied clinically and in therapeutic trials.

## Supplementary materials

Supplementary Materials_REVISED

## References

[1] Aziz I., Palsson O.S., Whitehead W.E. (2019). Epidemiology, clinical characteristics, and associations for Rome IV functional nausea and vomiting disorders in adults. Clin Gastroenterol Hepatol.

[2] Sperber A.D., Bangdiwala S.I., Drossman D.A. (2021). Worldwide prevalence and burden of functional gastrointestinal disorders, results of Rome Foundation Global Study. Gastroenterology.

[3] Sandler R.S., Everhart J.E., Donowitz M. (2002). The burden of selected digestive diseases in the United States. Gastroenterology.

[4] Stanghellini V., Chan F.K.L., Hasler W.L. (2016). Gastroduodenal disorders. Gastroenterology.

[5] Gharibans A.A., Calder S., Varghese C. (2022). Gastric dysfunction in patients with chronic nausea and vomiting syndromes defined by a noninvasive gastric mapping device. Sci Transl Med.

[6] Sperber A.D., Freud T., Aziz I. (2022). Greater overlap of Rome IV disorders of gut-brain interactions Leads to increased disease severity and poorer quality of life. Clin Gastroenterol Hepatol.

[7] Vanheel H., Carbone F., Valvekens L. (2017). Pathophysiological abnormalities in functional dyspepsia subgroups according to the Rome III criteria. Am J Gastroenterol.

[8] Lee H.-J., Lee S.Y., Kim J.H. (2010). Depressive mood and quality of life in functional gastrointestinal disorders: differences between functional dyspepsia, irritable bowel syndrome and overlap syndrome. Gen Hosp Psychiatry.

[9] Locke G. R., 3rd, Zinsmeister A.R., Fett S.L. (2005). Overlap of gastrointestinal symptom complexes in a US community. Neuro Gastroenterol Motil.

[10] Gharibans A.A., Coleman T.P., Mousa H. (2019). Spatial patterns from high-resolution electrogastrography correlate with severity of symptoms in patients with functional dyspepsia and gastroparesis. Clin Gastroenterol Hepatol.

[11] Wang W.J., Foong D., Calder S. (2024). Gastric alimetry expands patient phenotyping in gastroduodenal disorders compared with gastric emptying scintigraphy. Am J Gastroenterol.

[12] Xu W., Gharibans A.A., Calder S. (2023). Defining and phenotyping gastric abnormalities in long-term type 1 diabetes using a novel body surface gastric mapping device. Gastro Hep Adv.

[13] Sebaratnam G., Karulkar N., Calder S. (2022). Standardized system and App for continuous patient symptom logging in gastroduodenal disorders: design, implementation, and validation. Neuro Gastroenterol Motil.

[14] O'Grady G., Varghese C., Schamberg G. (2023). Principles and clinical methods of body surface gastric mapping: technical review. Neuro Gastroenterol Motil.

[15] Farré R., Vanheel H., Vanuytsel T. (2013). In functional dyspepsia, hypersensitivity to postprandial distention correlates with meal-related symptom severity. Gastroenterology.

[16] Drossman Douglas A., Tack J. Meet the ROME Foundation. https://theromefoundation.org/wp-content/uploads/Rome-Foundation_Meet-the-Foundation-booklet_2022-updated.pdf%20(2022-2023).

[17] Camilleri M., Chedid V. (2020). Actionable biomarkers: the key to resolving disorders of gastrointestinal function. Gut.

[18] Xu W., Wang T., Foong D. (2024). Characterization of gastric dysfunction after fundoplication using body surface gastric mapping. J Gastrointest Surg.

[19] Wang T.H.H., Varghese C., Calder S. (2023). Evaluation of gastric electrophysiology, symptoms and quality of life after pancreaticoduodenectomy. bioRxiv.

[20] Wang T.H.H., Varghese C., Calder S. (2023). Assessment of symptoms, quality of life and remnant gastric activity following gastric bypass using Gastric Alimetry®. bioRxiv.

[21] Vandenbroucke J.P., von Elm E., Altman D.G. (2007). Strengthening the reporting of observational studies in epidemiology (STROBE): explanation and elaboration. PLoS Med.

[22] Harer K.N., Pasricha P.J. (2016). Chronic unexplained nausea and vomiting or gastric neuromuscular dysfunction (GND)? An update on nomenclature, pathophysiology and treatment, and relationship to gastroparesis. Curr Treat Options Gastroenterol.

[23] Pasricha P.J., Grover M., Yates K.P. (2021). Functional dyspepsia and gastroparesis in tertiary care are interchangeable syndromes with common clinical and pathologic features. Gastroenterology.

[24] Angeli T.R., Cheng L.K., Du P. (2015). Loss of interstitial cells of cajal and patterns of gastric dysrhythmia in patients with chronic Unexplained nausea and vomiting. Gastroenterology.

[25] O'Grady G., Angeli T.R., Du P. (2012). Abnormal Initiation and conduction of slow-wave activity in gastroparesis, defined by high-resolution electrical mapping. Gastroenterology.

[26] Grover M., Bernard C.E., Pasricha P.J. (2012). Clinical-histological associations in gastroparesis: results from the gastroparesis clinical research consortium. Neuro Gastroenterol Motil.

[27] Carson D.A., Bhat S., Hayes T.C.L. (2021). Abnormalities on electrogastrography in nausea and vomiting syndromes: a systematic review, meta-analysis, and comparison to other gastric disorders. Dig Dis Sci.

[28] Gharibans A.A., Hayes T.C.L., Carson D.A. (2022). A novel scalable electrode array and system for non-invasively assessing gastric function using flexible electronics. Neuro Gastroenterol Motil.

[29] Varghese C., Schamberg G., Calder S. (2023). Normative values for body surface gastric mapping evaluations of gastric motility using gastric alimetry: spectral analysis. Am J Gastroenterol.

[30] Smout A.J.P.M., Van der Schee E.J., Grashuis J.L. (1980). What is measured in electrogastrography?. Dig Dis Sci.

[31] O’Grady G., Varghese C., Schamberg G. (2023). An Initial phenotype set for the assessment of gastroduodenal disorders with gastric Alimetry®. SSRN.

[32] Brzana R.J., Koch K.L., Bingaman S. (1998). Gastric myoelectrical activity in patients with gastric outlet obstruction and idiopathic gastroparesis. Am J Gastroenterol.

[33] Carson D.A., Robertson S., Wang T.H.H. (2023). The Impact and clinical implications of gastric surgery on the gastric conduction system. Foregut.

[34] Schamberg G., Varghese C., Uren E. (2023). Physiology-guided quantitative symptom analysis for gastroduodenal disorders. medRxiv.

[35] O'Grady G., Carbone F., Tack J. (2022). Gastric sensorimotor function and its clinical measurement. Neuro Gastroenterol Motil.

[36] Vanheel H., Vanuytsel T., Van Oudenhove L. (2013). Postprandial symptoms originating from the stomach in functional dyspepsia. Neuro Gastroenterol Motil.

[37] Ford A.C., Mahadeva S., Florencia Carbone M. (2020). Functional dyspepsia. Lancet.

[38] Abell T.L., Kedar A., Stocker A. (2021). Pathophysiology of gastroparesis syndromes includes anatomic and physiologic abnormalities. Dig Dis Sci.

[39] Furman D., Campisi J., Verdin E. (2019). Chronic inflammation in the etiology of disease across the life span. Nat. Med.

[40] Kroenke K., Spitzer R.L., Williams J.B.W. (2003). The Patient Health Questionnaire-2: validity of a two-item depression screener. Med Care.

[41] Spielberger C.D. State-trait anxiety inventory for adults. https://psycnet.apa.org/doiLanding?.

[42] Rentz A.M., Kahrilas P., Stanghellini V. (2004). Development and psychometric evaluation of the patient assessment of upper gastrointestinal symptom severity index (PAGI-SYM) in patients with upper gastrointestinal disorders. Qual Life Res.

[43] Revicki D.A., Rentz A.M., Dubois D. (2004). Gastroparesis Cardinal Symptom Index (GCSI): development and validation of a patient reported assessment of severity of gastroparesis symptoms. Qual Life Res.

[44] De la loge C., Trudeau E., Marquis P. (2004). Cross-cultural development and validation of a patient self-administered questionnaire to assess quality of life in upper gastrointestinal disorders: the PAGI-QOL©. Qual Life Res.

[45] Herdman M., Gudex C., Lloyd A. (2011). Development and preliminary testing of the new five-level version of EQ-5D (EQ-5D-5L). Qual Life Res.

[46] Calder S., Schamberg G., Varghese C. (2022). An automated artifact detection and rejection system for body surface gastric mapping. Neuro Gastroenterol Motil.

[47] Hasler W.L., Parkman H.P., Wilson L.A. (2010). Psychological dysfunction is associated with symptom severity but not disease etiology or degree of gastric retention in patients with gastroparesis. Am J Gastroenterol.

[48] Anisman H., Merali Z. (2002). Cytokines, stress, and depressive illness. Brain Behav Immun.

[49] Drossman D.A., Tack J., Ford A.C. (2018). Neuromodulators for functional gastrointestinal disorders (disorders of gut-brain interaction): A Rome Foundation working team Report. Gastroenterology.

[50] Drossman D.A., Tack J. (2022). Rome Foundation clinical diagnostic criteria for disorders of gut-brain interaction. Gastroenterology.

[51] Koloski N.A., Jones M., Kalantar J. (2012). The brain–gut pathway in functional gastrointestinal disorders is bidirectional: a 12-year prospective population-based study. Gut.

[52] Tack J., Carbone F., Holvoet L. (2014). The use of pictograms improves symptom evaluation by patients with functional dyspepsia. Aliment Pharmacol Ther.

[53] Gyawali C.P., Kahrilas P.J., Savarino E. (2018). Modern diagnosis of GERD: the Lyon consensus. Gut.

[54] Burns G., Carroll G., Mathe A. (2019). Evidence for local and systemic immune activation in functional dyspepsia and the irritable bowel syndrome: a systematic review. Am J Gastroenterol.

[55] Talley N.J., Walker M.M., Aro P. (2007). Non-ulcer dyspepsia and duodenal eosinophilia: an adult endoscopic population-based case-control study. Clin Gastroenterol Hepatol.

[56] Broeders B.W.L.C.M., Carbone F., Balsiger L.M. (2023). Review article: functional dyspepsia-a gastric disorder, a duodenal disorder or a combination of both?. Aliment Pharmacol Ther.

[57] Uezono Y., Miyano K., Tominaga K., Kusunoki H. (2018). Functional dyspepsia: evidences in pathophysiology and treatment.

[58] Mercado-Perez A., Beyder A. (2022). Gut feelings: mechanosensing in the gastrointestinal tract. Nat Rev Gastroenterol Hepatol.

[59] Hunt R.H., Camilleri M., Crowe S.E. (2015). The stomach in health and disease. Gut.

[60] Tack J., Verbeure W., Mori H. (2021). The gastrointestinal tract in hunger and satiety signalling. United European Gastroenterol J.

[61] Furness J.B., Clerc N., Kunze W.A. (2000). Memory in the enteric nervous system. Gut.

[62] Tack J., Corsetti M., Camilleri M. (2018). Plausibility criteria for putative pathophysiological mechanisms in functional gastrointestinal disorders: a consensus of experts. Gut.

[63] Park S.-Y., Acosta A., Camilleri M. (2017). Gastric motor dysfunction in patients with functional gastroduodenal symptoms. Am J Gastroenterol.

[64] Yadlapati R., Pandolfino J.E., Fox M.R. (2021). What is new in Chicago classification version 4.0?. Neuro Gastroenterol Motil.

[65] Wang W.J., Foong D., Calder S. (2023). Gastric Alimetry® expands patient phenotyping in gastroduodenal disorders compared to gastric emptying scintigraphy. Am J Gastroenterol.

