# Supplementary material for: A Standardized Classification Scheme for Gastroduodenal Disorder Evaluation Using the Gastric Alimetry System: Prospective Cohort Study
Source: Gastro Hep Adv. 2024 Sep 7;4(1):100547. doi: 10.1016/j.gastha.2024.09.002 (PMC11719321; doi:10.1016/j.gastha.2024.09.002)
Supplement: Supplementary Materials_REVISED [file mmc1.docx]

**Supplementary Materials**

**Supplementary Methods**

Missing data were excluded pairwise in regression models.

Gastric Alimetry Symptom Analysis Metrics and Methodology

*Meal Response.* The most studied characteristic of granular symptom data is the response of symptom severity to meal ingestion. Specifically, postprandial distress syndrome (PDS) is characterized by early satiation and/or excessive fullness, whereas epigastric pain syndrome (EPS), is associated with an increase or decrease in epigastric pain and/or burning following meal ingestion.^4^ Despite the importance of meal-related symptoms in gastroduodenal disorders, there is no agreed method for quantifying these changes. Therefore, to quantify the effect of meal ingestion on symptom severity, we define the *meal change* metric as the difference between the average symptom severities between the first hour postprandial and preprandial time windows. The *meal change* is thresholded to identify the **meal-induced** (*meal change* > 2) and **meal-relieved** (< -2) symptom phenotypes.

*Symptom Persistence.* It has been shown in patients with chronic nausea and vomiting disorders that those with normal Gastric Alimetry spectral analyses tend to have worse anxiety and/or depression than patients whose symptoms may be explained by gastric neuromuscular abnormalities.^29^ It was further recently demonstrated that, of patients with normal BSGM spectral analyses, pre-meal high symptom severity and persistence of high symptoms throughout the test was a phenotype that was highly associated with anxiety and/or depression.^12^ These results indicate that a high premeal symptom severity that persists through the test may be suggestive of disorders linked to the gut-brain axis. To quantify the symptom persistence, we define the *range* metric as the difference between the 95th and 5th percentile symptom severities. The **continuous** phenotype is identified by thresholding the *range* (i.e., little variation in symptoms in response to a meal or gastric activity) and the 5th percentile of symptom severity (*range* < 3 and 5th percentile > 2).

*Symptom-Amplitude Correlation.* A recent review has identified that a subset of patients exhibit symptoms that are tightly time-synchronized with the gastric amplitude, indicating that these symptoms may have a sensorimotor component and may be suggestive of disorders linked to visceral hypersensitivity and/or disordered gastric accommodation.^14,37^ To rigorously quantify this relationship, we define the *symptom-amplitude correlation* as the correlation coefficient between the symptom severity curve and gastric amplitude curve. The *symptom-amplitude correlation* is thresholded to identify the **sensorimotor** (*correlation* > 0.5) phenotype.

*Symptom-Amplitude Time Lag.* Patients may exhibit symptoms that occur before the onset of gastric contractile activity, suggesting symptoms may be related to a delayed onset of gastric mixing; and conversely, patients may experience symptoms occurring after the conclusion of a physiological gastric meal response, implying a pathology distal to the stomach.^14^ As a measure of the extent to which either of these patterns occur, we define the *symptom/amplitude time lag* as the average difference between the cumulative distribution functions of symptom and amplitude (-1 indicates all symptoms occurring before all gastric activity, and +1 all symptoms occurring after gastric activity). The *symptom/amplitude time lag* is thresholded to identify the **activity-relieved** (*lag* < -0.25) and **post-gastric** (> 0.25) phenotypes.

Symptom Metric Technical Development

*Preprocessing*

During a Gastric Alimetry test, patients are able to update their symptom severities at any point, with

reminders sent every 15 minutes. Before performing any quantitative analysis on a symptom severity

curve, the curve is linearly interpolated to have one value per minute, such that it matches the

timescale of the concurrently recorded gastric amplitude curve.

*Symptom Only Phenotypes*

The meal-induced, meal-relieved, and continuous phenotypes are computed for a given symptom

severity curve independently of the corresponding gastric amplitude.

The meal-induced and meal-relieved phenotypes are determined by thresholding the difference

between the average symptom severity in the entire preprandial period (~30 minutes) and the

average symptom severity in the first hour following meal completion (difference > 2 and < -2,

respectively).

The continuous phenotype is determined by applying a threshold to the range (the difference

between 95th and 5th percentiles of a given curve) and the 5th percentile. Thresholding the range (<

3) ensures that the symptom severity is not changing significantly throughout the test, while

thresholding the 5th percentile (> 2) ensures that the symptom is persisting at a non-negligible

severity (i.e., persistently low symptoms should not be deemed continuous). The 95th and 5th

percentiles are used in place of the maximum and minimum to ensure robustness to “misclicks” by the

patient. For example, if a patient accidentally updates a symptom to be very low and immediately

reverses it, that would affect the minimum but not the 5th percentile.

*Symptom/Amplitude Association Phenotypes*

The sensorimotor, activity-relieved, and post-gastric phenotypes are determined by comparing the

symptom curves to their associated time-synchronized gastric amplitude curves. Since each patient

reports multiple symptoms, a single gastric amplitude curve will be compared to five different

symptom severity curves. It is possible that a gastric amplitude curve will have missing values that

have been automatically removed by the Gastric Alimetry Algorithm due to excessive movement

artifacts. In such cases, the corresponding time points are also removed from the symptom severity

curves, as the two curves cannot be compared where there is data missing from one. Additionally, all

three of these phenotypes are only applied to cases where there is sufficient variation in both the

amplitude and symptom severity curves. For example, if a patient reports changes by a maximum of

one point on the 11-point likert scale, this may not be considered a significant enough change in

symptoms to evaluate its relationship to gastric activity. Formally, we only identify symptom/amplitude

association phenotypes when the standard deviation of the amplitude curve is > 10 μV and the

standard deviation of the symptom severity curve is > 0.5. This threshold was determined empirically with manual case review and sensitivity analyses to ensure alignment with clinical assessment of the sensorimotor pattern.

The sensorimotor phenotype is determined by thresholding the correlation coefficient between the

gastric amplitude and symptom severity curve (> 0.5). To account for the potential delay between the

experience of symptoms and reporting of symptoms, this correlation coefficient is calculated as the

maximum correlation coefficient allowing for a +/- 10 minute shift of the relative timing of the curves.

Specifically, the correlation coefficient is calculated 21 times (shift = -10 min, ..., 0 min, ... ,+10 min),

with the maximum correlation coefficient compared to the threshold.

The activity-relieved and post-gastric phenotypes are determined by comparing the average difference between cumulative distribution functions (CDFs) associated with the amplitude and

symptom severity curves (difference < -0.25 and > 0.25, respectively). The cumulative distribution

function for each curve serves to represent where in the test the amplitude or symptom burden is

concentrated. The steps to calculate the CDF for a given curve are: (1) normalize the curve so that it

sums to one, (2) take the cumulative sum of the normalized curve. As a result, the CDF is a function

that is increasing from zero at the beginning of the test to one at the end of the test, with the value at

each point in time representing the proportion of the total amplitude or symptom burden that has

already occurred. For example, if a particular symptom occurs primarily at the beginning of the test,

the CDF will start at zero, and quickly rise to be close to one. As a result, the average (across time)

difference between the CDFs for two curves will be positive if the concentration of gastric amplitude is

earlier than that of the symptoms and negative if it is later.

**Supplementary Tables**

**Table S1**: Comparison of symptom burden across overlapping ROME-IV categories​

| **Symptom** |  | **CNVS** | **EPS only** | **PDS only** | **EPS only+CNVS** | **PDS only+CNVS** | **PDS+EPS** | **PDS+EPS+CNVS** | ***P*** |
| --- | --- | --- | --- | --- | --- | --- | --- | --- | --- |
| Upper gut pain | Mean (SD) | 0.5 (0.4) | 2.8 (2.5) | 1.4 (2.4) | 2.7 (2.4) | 2.1 (2.1) | 1.3 (1.8) | 3.3 (2.4) | .002 |
| Nausea | Mean (SD) | 2.4 (2.0) | 0.4 (0.7) | 0.5 (0.9) | 3.0 (2.5) | 3.6 (2.7) | 0.6 (1.3) | 3.6 (2.6) | <.001 |
| Bloating | Mean (SD) | 0.7 (0.9) | 3.0 (2.9) | 3.4 (2.8) | 2.7 (3.2) | 3.2 (2.7) | 1.7 (1.5) | 3.4 (2.6) | .115 |
| Heartburn | Mean (SD) | 0.0 (0.0) | 0.8 (1.2) | 0.2 (0.5) | 1.3 (2.0) | 1.0 (1.8) | 0.5 (1.3) | 1.7 (2.4) | .075 |
| Stomach burn | Mean (SD) | 0.1 (0.1) | 1.6 (2.3) | 0.1 (0.4) | 1.1 (1.8) | 1.3 (1.8) | 0.7 (1.4) | 1.8 (2.4) | .069 |
| Excessively full | Mean (SD) | 1.1 (1.1) | 2.2 (2.3) | 3.2 (2.7) | 2.7 (2.7) | 4.2 (2.6) | 2.3 (2.4) | 4.3 (2.7) | <.001 |

EPS, epigastric pain syndrome; PDS, postprandial distress syndrome; CNVS, chronic nausea and vomiting syndromes.

**Table S2:** Spectral metrics across spectral phenotypes

| **Spectral Metric** | **Low rhythm stability / low amplitude** | **High stable amplitude** | **High Principal Frequency** | **Low Principal Frequency** | ***P*** |
| --- | --- | --- | --- | --- | --- |
| Principal Gastric Frequency | 3.0 (0.3) | 3.1 (0.2) | 3.5 (0.1) | 2.5 (0.1) | <.001 |
| BMI-adjusted amplitude | 25.0 (6.1) | 89.8 (15.5) | 47.9 (21.1) | 30.2 (3.9) | <.001 |
| GA-RI | 0.2 (0.1) | 0.6 (0.2) | 0.5 (0.2) | 0.5 (0.1) | <.001 |

GA-RI, Gastric Alimetry Rhythm Index; data are presented as mean (SD).

**Table S3**: Cross-tabulation of symptoms by symptom profile phenotype. A single symptom curve can have multiple pattern classifications. Percentages are by row.

|  | Sensorimotor | Continuous | Meal-induced | Post-gastric | Activity-relieved | Total |
| --- | --- | --- | --- | --- | --- | --- |
| Overall | 13 (14.8%) | 48 (54.5%) | 24 (27.3%) | 2 (2.3%) | 1 (1.1%) | 88 |
| Bloating | 15 (14.7%) | 37 (36.2%) | 48 (47.1%) | 2 (2.0%) | 0 (0.0%) | 102 |
| Heartburn | 0 (0.0%) | 40 (100%) | 0 (0.0%) | 0 (0.0%) | 0 (0.0%) | 40 |
| Nausea | 3 (10.3%) | 9 (31.0%) | 13 (44.8%) | 0 (0.0%) | 4 (13.8%) | 29 |
| Stomach burn | 8 (9.5%) | 27 (32.1%) | 45 (53.6%) | 0 (0.0%) | 4 (4.8%) | 84 |
| Upper gut pain | 6 (12.8%) | 16 (34.0%) | 20 (42.6%) | 3 (6.4%) | 2 (4.3%) | 47 |

**Table S4**: Prevalence of Gastric Alimetry™ profiles across Rome-IV cohorts

| **Phenotype** | **CNVS only (n = 4)** | **CNVS+FD (n = 165)** | **FD only (n = 41)** |
| --- | --- | --- | --- |
| **Spectral-Abn** | 2 (50.0) | 61 (37.0) | 16 (39.0) |
| **Sensorimotor** | - | 15 (9.1) | - |
| **Post-gastric** | - | 3 (1.8) | 1 (2.4) |
| **Activity-relieved** | - | 7 (4.2) | 1 (2.4) |
| **Continuous** | - | 31 (18.8) | 6 (14.6) |
| **Meal-relieved** | - | 2 (1.2) | 1 (2.4) |
| **Meal-induced** | - | 23 (13.9) | 5 (12.2) |
| **Other** | 2 (50.0) | 23 (13.9) | 11 (26.8) |

CNVS, chronic nausea and vomiting syndromes; FD, functional dyspepsia.

**Table S5**: Association between Gastric Alimetry™ phenotypes and symptoms (GCSI, PAGI-SYM), depression (PHQ-2), anxiety (State-STAI), and quality of life (EQ-5D, PAGI-QOL)

|  | | | | | | | | | | | | | | | | | | |
| --- | --- | --- | --- | --- | --- | --- | --- | --- | --- | --- | --- | --- | --- | --- | --- | --- | --- | --- |
| **Characteristic** | GCSI | | | PHQ-2 | | | State STAI | | | PAGI-QOL | | | PAGI-SYM | | | EQ5D | | |
|  | **exp(β)** | **95% CI** | ***P*** | **exp(β)** | **95% CI** | ***P*** | **exp(β)** | **95% CI** | ***P*** | **exp(β)** | **95% CI** | ***P*** | **exp(β)** | **95% CI** | ***P*** | **exp(β)** | **95% CI** | ***P*** |
| Age | 0.99 | 0.98, 0.99 | <.001 | 0.98 | 0.97, 1.00 | .025 | 1 | 0.99, 1.00 | .15 | 1.01 | 1.00, 1.02 | .048 | 0.99 | 0.98, 0.99 | <.001 | 1 | 1.00, 1.00 | .4 |
| Sex |  |  |  |  |  |  |  |  |  |  |  |  |  |  |  |  |  |  |
| Female | — | — |  | — | — |  | — | — |  | — | — |  | — | — |  | — | — |  |
| Male | 0.6 | 0.44, 0.82 | .002 | 0.94 | 0.50, 1.78 | .9 | 1.1 | 0.96, 1.26 | .2 | 1.24 | 0.90, 1.70 | .2 | 0.67 | 0.51, 0.88 | .004 | 1.03 | 0.96, 1.11 | .4 |
| BMI | 1.01 | 0.99, 1.03 | .2 | 1.03 | 0.99, 1.07 | .2 | 1.01 | 1.00, 1.02 | .12 | 0.99 | 0.97, 1.01 | .2 | 1.02 | 1.00, 1.04 | .032 | 1 | 0.99, 1.00 | .4 |
| Gastric Alimetry™ Phenotype |  |  |  |  |  |  |  |  |  |  |  |  |  |  |  |  |  |  |
| Other | — | — |  | — | — |  | — | — |  | — | — |  | — | — |  | — | — |  |
| Continuous | 1.93 | 1.28, 2.93 | .002 | 1.97 | 0.85, 4.59 | .12 | 1.19 | 0.99, 1.43 | .07 | 0.53 | 0.35, 0.82 | .004 | 1.92 | 1.34, 2.76 | <.001 | 0.8 | 0.71, 0.89 | <.001 |
| Meal-relieved | 1.09 | 0.38, 3.12 | .9 | 1.12 | 0.13, 9.51 | >.9 | 1.43 | 0.88, 2.34 | .2 | 1.36 | 0.46, 4.05 | .6 | 1.1 | 0.44, 2.76 | .8 | 0.98 | 0.78, 1.23 | .9 |
| Activity-relieved | 1.37 | 0.69, 2.72 | .4 | 2.3 | 0.57, 9.30 | .2 | 1.24 | 0.91, 1.67 | .2 | 0.41 | 0.20, 0.84 | .015 | 1.49 | 0.82, 2.72 | .2 | 0.92 | 0.76, 1.11 | .4 |
| Meal-induced | 1.76 | 1.13, 2.73 | .013 | 3.35 | 1.35, 8.31 | .01 | 1.21 | 1.00, 1.46 | .047 | 0.45 | 0.28, 0.71 | <.001 | 1.5 | 1.02, 2.21 | .04 | 0.81 | 0.73, 0.91 | <.001 |
| Post-gastric | 1.28 | 0.50, 3.28 | .6 | 4.22 | 0.63, 28.1 | .14 | 1.41 | 0.98, 2.03 | .068 | 0.27 | 0.10, 0.70 | .008 | 1.35 | 0.60, 3.06 | .5 | 0.82 | 0.65, 1.04 | .11 |
| Sensorimotor | 1.97 | 1.15, 3.39 | .014 | 1.09 | 0.35, 3.35 | .9 | 1.03 | 0.81, 1.31 | .8 | 0.72 | 0.41, 1.26 | .3 | 1.62 | 1.01, 2.60 | .046 | 0.9 | 0.79, 1.03 | .12 |
| Spectral-Abn | 1.6 | 1.13, 2.28 | .009 | 1.32 | 0.64, 2.71 | .5 | 1 | 0.86, 1.16 | >.9 | 0.69 | 0.48, 0.99 | .047 | 1.52 | 1.12, 2.06 | .009 | 0.92 | 0.84, 1.01 | .076 |
|  | | | | | | | | | | | | | | | | | | |

**Table S6:** Association between Individual symptoms and association between BSGM classification and individual symptom classifications and chronic symptoms, depression, anxiety, and quality of life

| Upper gut pain | | | | | | | | | | | | | | | |
| --- | --- | --- | --- | --- | --- | --- | --- | --- | --- | --- | --- | --- | --- | --- | --- |
| **Characteristic** | GCSI | | | PHQ-2 | | | State STAI | | | PAGI-QOL | | | PAGI-SYM | | |
|  | **exp(β)** | **95% CI***^1^* | ***P*** | **exp(β)** | **95% CI***^1^* | ***P*** | **exp(β)** | **95% CI***^1^* | ***P*** | **exp(β)** | **95% CI***^1^* | ***P*** | **exp(β)** | **95% CI***^1^* | ***P*** |
| Age | 0.98 | 0.98, 0.99 | <.001 | 0.98 | 0.97, 1.00 | .023 | 1 | 0.99, 1.00 | .068 | 1.01 | 1.00, 1.02 | .021 | 0.98 | 0.98, 0.99 | <.001 |
| Sex |  |  |  |  |  |  |  |  |  |  |  |  |  |  |  |
| Female | — | — |  | — | — |  | — | — |  | — | — |  | — | — |  |
| Male | 0.58 | 0.42, 0.80 | .001 | 0.93 | 0.49, 1.76 | .8 | 1.08 | 0.94, 1.24 | .3 | 1.25 | 0.91, 1.73 | .2 | 0.66 | 0.50, 0.86 | .003 |
| BMI | 1.01 | 0.99, 1.04 | .2 | 1.03 | 0.98, 1.07 | .2 | 1.01 | 1.00, 1.02 | .2 | 0.99 | 0.97, 1.01 | .2 | 1.02 | 1.00, 1.04 | .019 |
| Upper Gut Pain Profile |  |  |  |  |  |  |  |  |  |  |  |  |  |  |  |
| Other | — | — |  | — | — |  | — | — |  | — | — |  | — | — |  |
| Continuous | 1.36 | 0.84, 2.20 | .2 | 0.99 | 0.39, 2.55 | >.9 | 0.94 | 0.76, 1.18 | .6 | 0.7 | 0.43, 1.14 | .2 | 1.74 | 1.15, 2.63 | .009 |
| Meal-relieved | 1.12 | 0.18, 6.82 | >.9 | 33.6 | 0.97, 1,168 | .054 | 1.45 | 0.74, 2.86 | .3 | 0.68 | 0.11, 4.21 | .7 | 1.44 | 0.31, 6.76 | .6 |
| Activity-relieved | 1.22 | 0.42, 3.54 | .7 | 2.4 | 0.29, 19.5 | .4 | 1.03 | 0.69, 1.55 | .9 | 0.37 | 0.13, 1.10 | .076 | 1.42 | 0.57, 3.55 | .5 |
| Meal-induced | 1.29 | 0.82, 2.04 | .3 | 2.59 | 1.03, 6.52 | .045 | 1.15 | 0.95, 1.40 | .2 | 0.44 | 0.28, 0.70 | <.001 | 1.48 | 1.00, 2.19 | .05 |
| Post-gastric | 0.93 | 0.32, 2.69 | .9 | 0.65 | 0.08, 5.22 | .7 | 0.96 | 0.64, 1.43 | .8 | 0.42 | 0.14, 1.22 | .11 | 1.1 | 0.45, 2.74 | .8 |
| Sensorimotor | 1.29 | 0.45, 3.70 | .6 | 0.18 | 0.02, 1.44 | .11 | 0.67 | 0.41, 1.09 | .11 | 1.93 | 0.67, 5.61 | .2 | 1.45 | 0.59, 3.59 | .4 |
| Spectral-Abn | 1.18 | 0.89, 1.56 | .3 | 0.87 | 0.50, 1.53 | .6 | 0.89 | 0.79, 1.00 | .06 | 0.9 | 0.68, 1.20 | .5 | 1.25 | 0.98, 1.59 | .071 |
| Nausea | | | | | | | | | | | | | | | |
| **Characteristic** | GCSI | | | PHQ-2 | | | State STAI | | | PAGI-QOL | | | PAGI-SYM | | |
|  | **exp(β)** | **95% CI***^1^* | ***P*** | **exp(β)** | **95% CI***^1^* | ***P*** | **exp(β)** | **95% CI***^1^* | ***P*** | **exp(β)** | **95% CI***^1^* | **p** | **exp(β)** | **95% CI***^1^* | ***P*** |
| Age | 0.99 | 0.98, 0.99 | <.001 | 0.98 | 0.97, 1.00 | .028 | 1 | 0.99, 1.00 | .12 | 1.01 | 1.00, 1.02 | .055 | 0.99 | 0.98, 0.99 | <.001 |
| Sex |  |  |  |  |  |  |  |  |  |  |  |  |  |  |  |
| Female | — | — |  | — | — |  | — | — |  | — | — |  | — | — |  |
| Male | 0.62 | 0.45, 0.84 | .002 | 1.02 | 0.54, 1.95 | >.9 | 1.1 | 0.96, 1.26 | .2 | 1.2 | 0.87, 1.66 | .3 | 0.65 | 0.49, 0.85 | .002 |
| BMI | 1.01 | 0.99, 1.03 | .3 | 1.03 | 0.98, 1.07 | .2 | 1.01 | 1.00, 1.01 | .2 | 0.99 | 0.97, 1.01 | .3 | 1.02 | 1.00, 1.04 | .024 |
| Nausea Profile |  |  |  |  |  |  |  |  |  |  |  |  |  |  |  |
| Other | — | — |  | — | — |  | — | — |  | — | — |  | — | — |  |
| Continuous | 2.38 | 1.50, 3.79 | <.001 | 3.26 | 1.24, 8.60 | .018 | 1.33 | 1.08, 1.63 | .007 | 0.65 | 0.40, 1.07 | .091 | 1.59 | 1.05, 2.42 | .031 |
| Meal-relieved | 2.56 | 0.75, 8.80 | .14 | 0.85 | 0.07, 11.1 | >.9 | 1.35 | 0.83, 2.19 | .2 | 1.02 | 0.27, 3.79 | >.9 | 1.89 | 0.62, 5.74 | .3 |
| Activity-relieved | 0.98 | 0.29, 3.35 | >.9 | 2.57 | 0.20, 33.1 | .5 | 1.13 | 0.58, 2.21 | .7 | 0.67 | 0.18, 2.48 | .6 | 1.08 | 0.36, 3.26 | .9 |
| Meal-induced | 1.91 | 1.32, 2.75 | <.001 | 1.78 | 0.83, 3.84 | .14 | 1.12 | 0.96, 1.31 | .2 | 0.46 | 0.31, 0.68 | <.001 | 1.39 | 1.00, 1.93 | .055 |
| Sensorimotor | 2.65 | 0.78, 9.08 | .12 | 0.49 | 0.04, 6.29 | .6 | 0.92 | 0.57, 1.49 | .7 | 1.83 | 0.49, 6.76 | .4 | 2.18 | 0.72, 6.61 | .2 |
| Spectral-Abn | 1.43 | 1.09, 1.88 | .012 | 1.02 | 0.57, 1.82 | >.9 | 0.94 | 0.84, 1.07 | .4 | 0.87 | 0.65, 1.16 | .4 | 1.26 | 0.98, 1.61 | .07 |
| Bloating | | | | | | | | | | | | | | | |
| **Characteristic** | GCSI | | | PHQ-2 | | | State STAI | | | PAGI-QOL | | | PAGI-SYM | | |
|  | **exp(β)** | **95% CI***^1^* | ***P*** | **exp(β)** | **95% CI***^1^* | ***P*** | **exp(β)** | **95% CI***^1^* | ***P*** | **exp(β)** | **95% CI***^1^* | ***P*** | **exp(β)** | **95% CI***^1^* | ***P*** |
| Age | 0.98 | 0.98, 0.99 | <.001 | 0.98 | 0.97, 1.00 | .011 | 1 | 0.99, 1.00 | .064 | 1.01 | 1.00, 1.02 | .021 | 0.98 | 0.98, 0.99 | <.001 |
| Sex |  |  |  |  |  |  |  |  |  |  |  |  |  |  |  |
| Female | — | — |  | — | — |  | — | — |  | — | — |  | — | — |  |
| Male | 0.58 | 0.42, 0.79 | <.001 | 0.89 | 0.47, 1.69 | .7 | 1.08 | 0.95, 1.24 | .3 | 1.32 | 0.95, 1.84 | .1 | 0.64 | 0.49, 0.84 | .002 |
| BMI | 1.01 | 1.0, 1.03 | .15 | 1.03 | 0.99, 1.07 | .13 | 1.01 | 1.00, 1.02 | .14 | 0.99 | 0.97, 1.01 | .2 | 1.02 | 1.01, 1.04 | .011 |
| Bloating Profile |  |  |  |  |  |  |  |  |  |  |  |  |  |  |  |
| Other | — | — |  | — | — |  | — | — |  | — | — |  | — | — |  |
| Continuous | 1.78 | 1.14, 2.78 | .012 | 1.19 | 0.48, 2.93 | .7 | 1.09 | 0.90, 1.32 | .4 | 0.73 | 0.46, 1.18 | .2 | 1.62 | 1.10, 2.40 | .016 |
| Meal-induced | 1.2 | 0.82, 1.76 | .4 | 0.76 | 0.34, 1.67 | .5 | 0.95 | 0.80, 1.13 | .6 | 0.74 | 0.50, 1.12 | .2 | 1.21 | 0.86, 1.69 | .3 |
| Post-gastric | 0.56 | 0.09, 3.29 | .5 | 47.4 | 1.32, 1,711 | .036 | 1.95 | 0.99, 3.83 | .056 | 0.36 | 0.05, 2.39 | .3 | 0.98 | 0.21, 4.63 | >.9 |
| Sensorimotor | 1.37 | 0.68, 2.76 | .4 | 0.55 | 0.13, 2.28 | .4 | 0.98 | 0.73, 1.30 | .9 | 0.97 | 0.46, 2.03 | >.9 | 1.05 | 0.57, 1.94 | .9 |
| Spectral-Abn | 1.24 | 0.93, 1.66 | .14 | 0.75 | 0.41, 1.35 | .3 | 0.89 | 0.78, 1.01 | .066 | 0.96 | 0.71, 1.30 | .8 | 1.21 | 0.94, 1.56 | .13 |
| Heartburn | | | | | | | | | | | | | | | |
| **Characteristic** | GCSI | | | PHQ-2 | | | State STAI | | | PAGI-QOL | | | PAGI-SYM | | |
|  | **exp(β)** | **95% CI***^1^* | ***P*** | **exp(β)** | **95% CI***^1^* | ***P*** | **exp(β)** | **95% CI***^1^* | ***P*** | **exp(β)** | **95% CI***^1^* | ***P*** | **exp(β)** | **95% CI***^1^* | ***P*** |
| Age | 0.98 | 0.98, 0.99 | <.001 | 0.98 | 0.97, 1.00 | .03 | 1 | 0.99, 1.00 | .14 | 1.01 | 1.00, 1.02 | .051 | 0.98 | 0.98, 0.99 | <.001 |
| Sex |  |  |  |  |  |  |  |  |  |  |  |  |  |  |  |
| Female | — | — |  | — | — |  | — | — |  | — | — |  | — | — |  |
| Male | 0.57 | 0.42, 0.78 | <.001 | 0.9 | 0.48, 1.70 | .7 | 1.09 | 0.95, 1.24 | .2 | 1.31 | 0.95, 1.81 | .1 | 0.63 | 0.48, 0.83 | .001 |
| BMI | 1.01 | 0.99, 1.04 | .2 | 1.02 | 0.98, 1.06 | .3 | 1 | 1.00, 1.01 | .4 | 0.99 | 0.97, 1.01 | .5 | 1.02 | 1.00, 1.04 | .033 |
| Heartburn Profile |  |  |  |  |  |  |  |  |  |  |  |  |  |  |  |
| Other | — | — |  | — | — |  | — | — |  | — | — |  | — | — |  |
| Continuous | 0.96 | 0.50, 1.86 | .9 | 1.57 | 0.42, 5.84 | .5 | 1.2 | 0.91, 1.60 | .2 | 0.57 | 0.29, 1.13 | .11 | 1.29 | 0.73, 2.28 | .4 |
| Activity-relieved | 1.1 | 0.38, 3.17 | .9 | 9.32 | 1.15, 75.8 | .038 | 1.59 | 0.98, 2.58 | .064 | 0.3 | 0.10, 0.90 | .032 | 1.31 | 0.53, 3.26 | .6 |
| Meal-induced | 1.28 | 0.72, 2.27 | .4 | 1.75 | 0.56, 5.50 | .3 | 1.08 | 0.85, 1.36 | .5 | 0.67 | 0.37, 1.22 | .2 | 1.6 | 0.97, 2.64 | .065 |
| Spectral-Abn | 1.1 | 0.84, 1.44 | .5 | 0.87 | 0.51, 1.50 | .6 | 0.9 | 0.81, 1.01 | .085 | 0.99 | 0.75, 1.31 | >.9 | 1.15 | 0.91, 1.44 | .3 |
| Stomach burn | | | | | | | | | | | | | | | |
| **Characteristic** | GCSI | | | PHQ-2 | | | State STAI | | | PAGI-QOL | | | PAGI-SYM | | |
|  | **exp(β)** | **95% CI***^1^* | ***P*** | **exp(β)** | **95% CI***^1^* | ***P*** | **exp(β)** | **95% CI***^1^* | ***P*** | **exp(β)** | **95% CI***^1^* | ***P*** | **exp(β)** | **95% CI***^1^* | ***P*** |
| Age | 0.98 | 0.98, 0.99 | <.001 | 0.98 | 0.97, 1.00 | .021 | 1 | 0.99, 1.00 | .1 | 1.01 | 1.00, 1.02 | .039 | 0.98 | 0.98, 0.99 | <.001 |
| Sex |  |  |  |  |  |  |  |  |  |  |  |  |  |  |  |
| Female | — | — |  | — | — |  | — | — |  | — | — |  | — | — |  |
| Male | 0.62 | 0.45, 0.84 | .003 | 0.95 | 0.49, 1.81 | .9 | 1.08 | 0.94, 1.24 | .3 | 1.26 | 0.90, 1.76 | .2 | 0.68 | 0.52, 0.90 | .006 |
| BMI | 1.01 | 0.99, 1.03 | .3 | 1.02 | 0.98, 1.06 | .4 | 1 | 1.00, 1.01 | .3 | 0.99 | 0.97, 1.01 | .3 | 1.02 | 1.00, 1.04 | .037 |
| Stomach Burn Profile |  |  |  |  |  |  |  |  |  |  |  |  |  |  |  |
| Other | — | — |  | — | — |  | — | — |  | — | — |  | — | — |  |
| Continuous | 1.47 | 0.79, 2.73 | .2 | 1.88 | 0.54, 6.59 | .3 | 1.18 | 0.93, 1.50 | .2 | 0.78 | 0.41, 1.50 | .5 | 1.73 | 1.02, 2.95 | .045 |
| Meal-relieved | 0.14 | 0.02, 0.86 | .035 | 0.11 | 0.00, 4.19 | .2 |  |  |  | 6.11 | 0.91, 40.9 | .064 | 0.15 | 0.03, 0.69 | .016 |
| Activity-relieved | 0.82 | 0.23, 2.90 | .8 | 0.58 | 0.04, 7.67 | .7 | 0.89 | 0.45, 1.77 | .8 | 0.96 | 0.25, 3.70 | >.9 | 0.97 | 0.32, 2.89 | >.9 |
| Meal-induced | 1.2 | 0.75, 1.92 | .4 | 1.37 | 0.53, 3.54 | .5 | 1.06 | 0.87, 1.29 | .5 | 0.73 | 0.45, 1.20 | .2 | 1.43 | 0.95, 2.14 | .085 |
| Post-gastric | 1.39 | 0.49, 3.95 | .5 | 0.41 | 0.03, 5.37 | .5 | 0.78 | 0.48, 1.27 | .3 | 1.03 | 0.34, 3.10 | >.9 | 1.25 | 0.51, 3.08 | .6 |
| Sensorimotor | 3.02 | 0.85, 10.7 | .09 | 5.02 | 0.38, 66.2 | .2 | 1.14 | 0.70, 1.85 | .6 | 0.64 | 0.17, 2.44 | .5 | 2.21 | 0.74, 6.59 | .2 |
| Spectral-Abn | 1.13 | 0.87, 1.48 | .4 | 0.82 | 0.47, 1.44 | .5 | 0.9 | 0.80, 1.01 | .081 | 1.04 | 0.78, 1.38 | .8 | 1.16 | 0.92, 1.47 | .2 |
| *^1^* CI = Confidence Interval | | | | | | | | | | | | | | | |

**Supplementary Figures**

**Figure S1**: Overlap between ROME-IV defined functional dyspepsia (postprandial distress syndrome (PDS) and epigastric pain syndrome (EPS) subtypes) and chronic nausea and vomiting syndromes (CNVS).

**
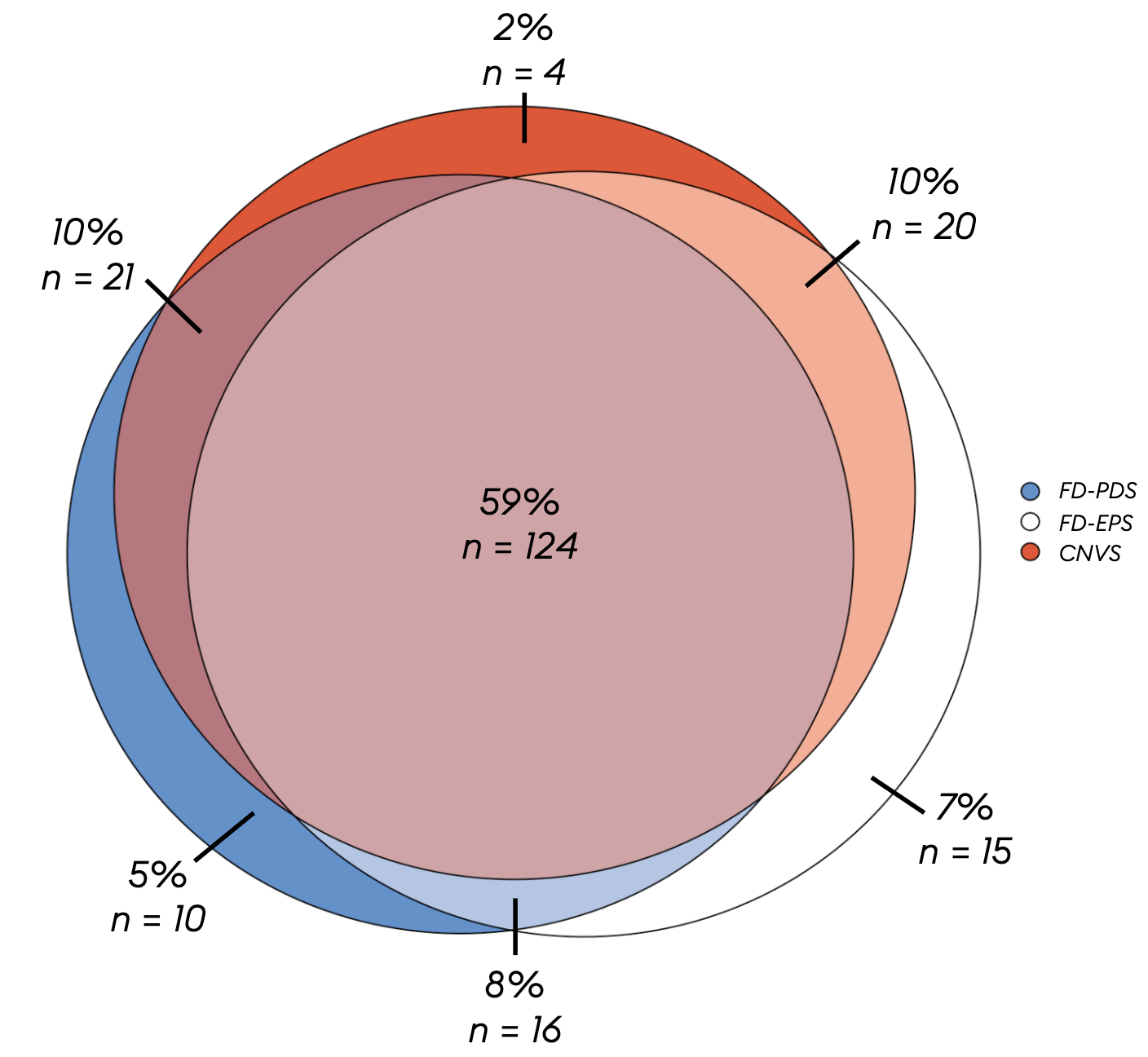
**

**Figure S2**: Comparison of symptom burden across overlapping Rome-IV categories

**
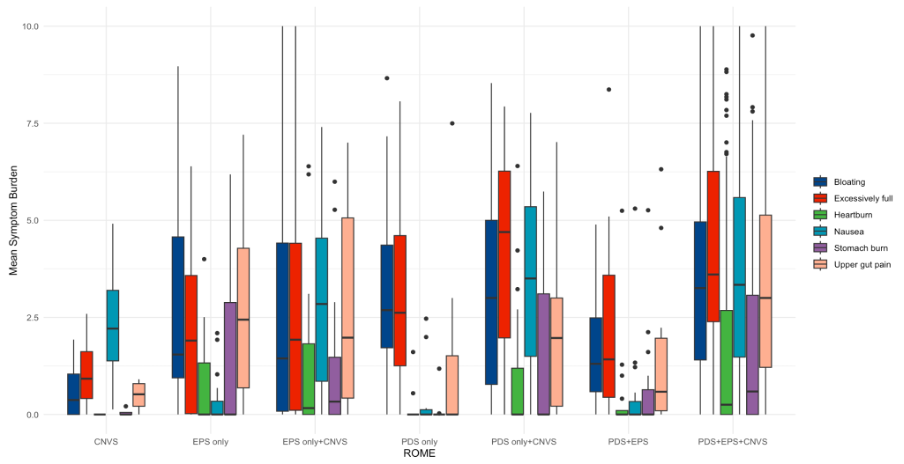
**

**Figure S3:** Spider plot of average symptom burden (0-10 with lines at interval of 2). A) Chronic nausea and vomiting syndromes (CNVS) + functional dyspepsia (FD) cohort, B) FD only cohort, C) CNVS only cohort, D) spectral-abnormal (Abn) cohort, E) Amplitude-related symptom pattern cohort, and F) amplitude-independent symptom pattern cohort. This plot highlights that symptoms alone do not separate groups, but better differentiation of groups with overlapping symptoms is facilitated by mechanism-based symptom profiling patterns.


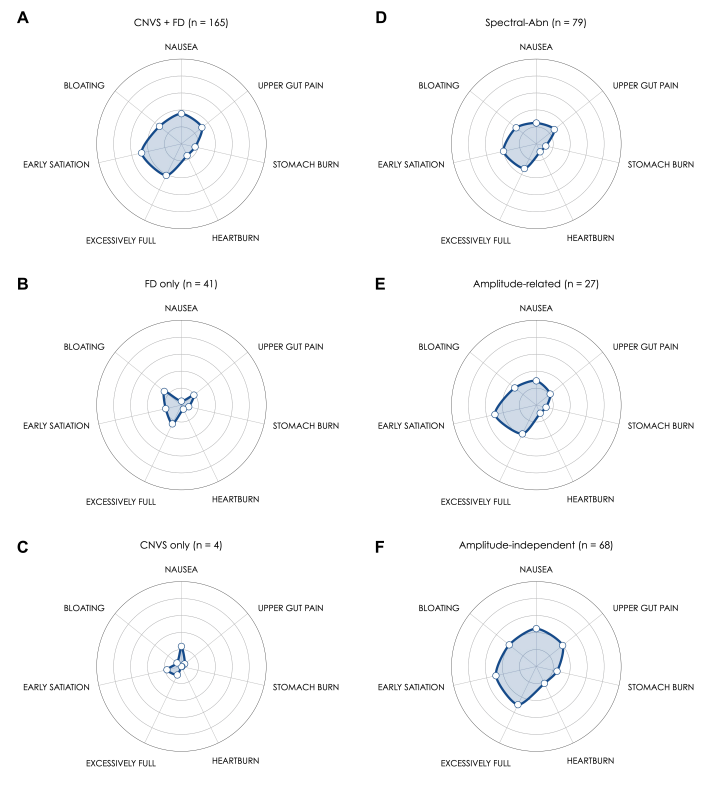


**Figure S4:** A- B) Average spectrogram, median amplitude curve (IQR shaded), and median average symptom burden (IQR shaded) for those with the post-gastric symptom patterns (n = 9) and the activity-relieved symptom patterns (n = 15). C – E) Median (IQR shaded) cumulative distribution functions for symptom and amplitude curves among symptoms with A) post-gastric pattern, B) activity-relieved pattern, and C) all other patterns that do not meet a lag-based criterion. *Cumulative distribution function, CDF.*

**
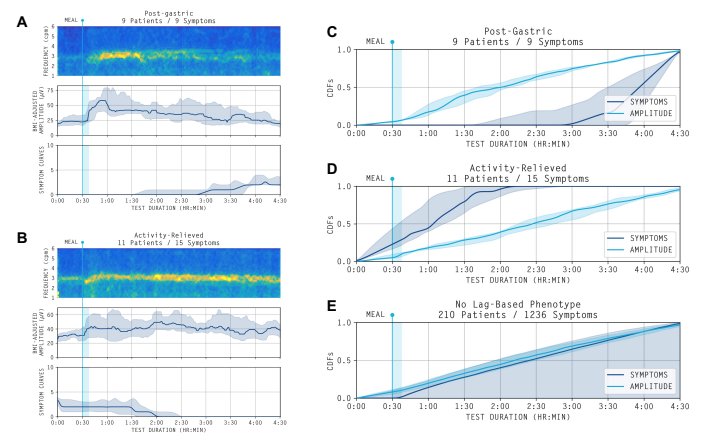
**

**Figure S5:** Average symptom burden plots for symptom patterns defined independently to gastric amplitude; A) continuous pattern, B) meal-relieved pattern, C) meal-induced pattern.

**
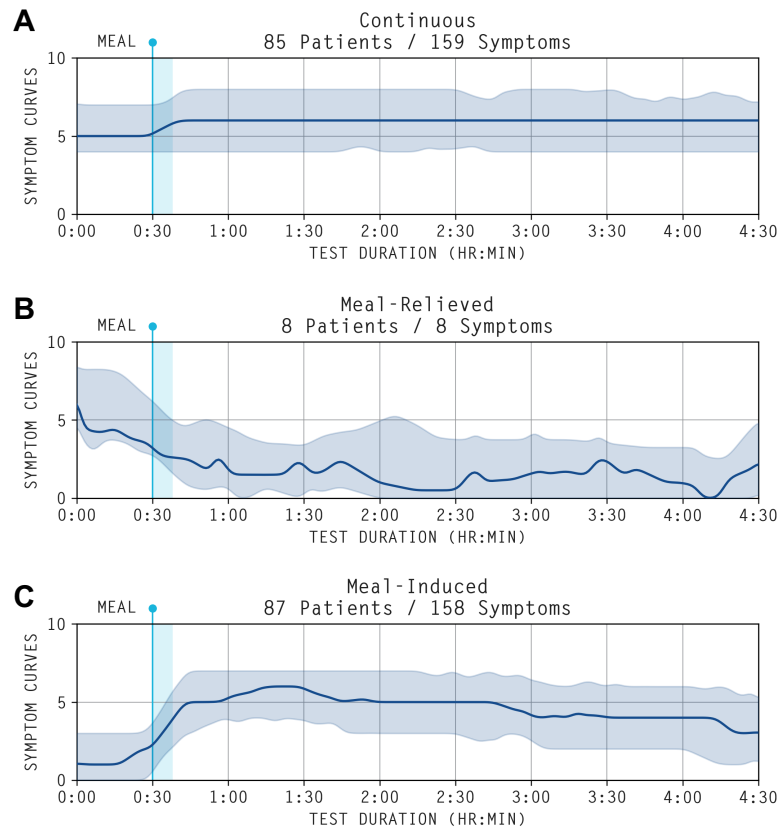
**
